# Reprogramming Immunodeficiency in Lung Metastases via PD-L1 siRNA Delivery and Antigen Capture of Nanosponge-Mediated Dendritic Cell Modulation

**DOI:** 10.1021/acsnano.5c05395

**Published:** 2025-07-05

**Authors:** Thi My Hue Huynh, Pin-Xuan Huang, Kang-Li Wang, Ngoc-Tri Tran, Hoi Man Iao, Wan-Chi Pan, Yun-Hsuan Chang, Hui-Wen Lien, Alan Yueh-Luen Lee, Tsu-Chin Chou, Wen-Hsuan Chiang, Shang-Hsiu Hu

**Affiliations:** † Department of Biomedical Engineering and Environmental Sciences, 34881 National Tsing Hua University, Hsinchu 300044, Taiwan; ‡ National Institute of Cancer Research, National Health Research Institutes, Miaoli County 35053, Taiwan; § Institute of Analytical and Environmental Sciences, National Tsing Hua University, Hsinchu 300044, Taiwan; ∥ Department of Chemical Engineering, 34916 National Chung Hsing University, Taichung 402, Taiwan

**Keywords:** reprogramming immunodeficiency, lung metastases, gene delivery, immunotherapy, nanomedicine

## Abstract

Infiltration of cytotoxic T lymphocytes into hypovascular metastases offers significant potential for suppressing even the most intractable metastatic tumors, with dendritic cells (DCs) serving as pivotal initiators of antitumor immune responses during immunotherapy. However, the immune-privileged nature of hypovascular lung metastases combined with the inherently low immunogenicity of tumor clusters poses substantial barriers to effective lymphocyte recruitment. Here, a pH-responsive lung metastatic-targeted catalyst containing the tumor penetration polymer (TP)/solid lipids (SL)-coated Prussian blue (TP-SL@PB)-enhanced PD-L1 siRNA delivery and self-cascade antigen capture is developed for reprogramming immunodeficiency. Intravenously injected TP-SL@PB accumulated in the blood vessel-poor lung metastases via the organ-selective targeting and charge conversion of TP. In tumor clusters, SL@PB exerts catalytic and lysosomal escape effects, easily enhancing siRNA delivery and thus downregulating PD-L1. Catalysis also promotes the release of tumor-associated antigens (TAAs), including neoantigens and damage-associated molecular patterns. Subsequently, both positive TPs and SLs on PBs can act as antigen sponges to deliver TAAs to dendritic cells, thereby inducing long-term immune activation. TP-SL@PB acts as a hypovascularized lung metastasis-penetrating catalytic nanosponge, selecting T cells to infiltrate metastases and enhance immunotherapy.

## Graphical abstract

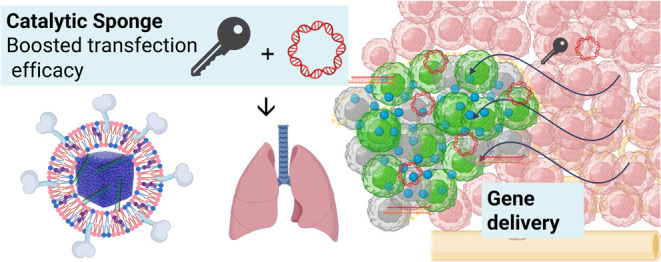

## 1 Introduction

Cancer immunotherapy has gradually gained prominence in clinical treatment of lung cancer patients by recruiting the host immune system.^1,2^ Despite breakthroughs in immunotherapy, a large proportion of cancer patients remain resistant to the current treatment options, with the effectiveness of these inhibitors often falling short of expectations.^3^ The difficulty stems from the nonspecific binding in tumor microenvironments hindering T-cell activity, especially in hypovascularized metastases. Even with PD-1/PD-L1 inhibition, continuous PD-L1 production by cancer cells suppresses T-cell function, allowing cancer cells to evade immune surveillance.^4^ This problem is particularly acute in small metastases (usually <100 mm^3^) because inadequate vascularization limits the physical interactions between T cells and cancer cells.^5,6^ Immune privilege, hindering T-cell recognition, inhibits the activation of antigen-presenting cells (APCs).^7,8^ Therefore, downregulation of PD-L1 and activation of tumor-specific T lymphocytes are crucial for the effectiveness of immunotherapy against secondary tumor invasion.

To address this issue, a simple strategy is to induce accessibility of T-cell-mediated immunotherapy, including immunosuppressive metabolic crosstalk between tumor and immune cells, such as downregulation of PD-L1.^6,9,10^ Reprogramming of tumor microenvironments (TME) metabolism regulates tumor progression and alters its immune sensitivity.^11,12^ In this regard, short interfering RNA (siRNA)-based therapies can inhibit specific gene expression and enhance immune responses against tumors by activating Toll-like receptors in immunotherapy.^13–15^ Advances in clinical gene delivery, Moderna’s COVID-19 vaccine highlights potential of siRNA therapeutics.^16,17^ The first major clinical success of siRNA therapy was achieved using the FDA-approved lipid nanoparticle (LNP)-based siRNA delivery system, patisiran, to liver to silence specific proteins.^18,19^ Although this is an important milestone, the expansion of siRNA therapy to other tissues still faces obstacles due to rapid clearance, degradation, and off-target effects of siRNA, especially in metastatic tumors with poor vascularity.^20,21^ Furthermore, it has difficulty penetrating the cell membrane and reaching the cytoplasm in the cells of specific organs.^22^ Therefore, the development of delivery vehicles that can both protect siRNA from degradation *in vivo* and ensure its delivery to the cytoplasm of hypovascular tissues is crucial for siRNA-based cancer therapy.

Inspired by these observations, organ-selective and permeable nanomedicines have the potential to enhance siRNA delivery of hypovascular metastases and T-cell-mediated immunotherapy.^23^ Recently, nanomedicines significantly boost T-cell uptake efficiency, ensuring that siRNA reaches its intracellular targets via improving cellular uptake and promoting endosomal escape.^24,25^ However, most intravenously administered nanomedicines are rapidly engulfed and eliminated by the reticuloendothelial system in animal models, leading to strong liver and spleen accumulation.^26^ To address this challenge, engineered delivery systems incorporating lipids and tumor-targeted polymers onto nanoparticles with “stealth” coatings have been developed to improve circulation times and reduce nonspecific binding.^27,28^ For organ-selective targeting, positively charged lipids have demonstrated promising results, enhancing lung delivery and achieving approximately 50% lung accumulation.^29,30^ Moreover, the weakly acidic tumor microenvironment has inspired the creation of pH-responsive polymers capable of charge conversion, enabling improved tumor targeting.^31,32^ At tumor sites, positively charged molecules exhibit membrane-disrupting abilities under acidic conditions (pH 6.4–6.8).^33,34^ In this context, pH-sensitive nanoparticles enhance tumor permeability and prolong the circulation of polyzwitterionic drug conjugates.

Regulating tumor-associated antigen (TAA) presentation coupled with antigen transport in activating immune response pathways in the tumor has great potential to amplify the *in situ* vaccination effect. Chemodynamic therapy (CDT) is a promising cancer treatment strategy that exploits advantage of tumor-specific biochemical properties to produce reactive oxygen species (ROS) through the Fenton reaction. This reaction involves the catalytic conversion of H_2_O_2_ into highly reactive hydroxyl radicals (^•^OH) in the presence of ferrous ions (Fe^2+^), leading to oxidative stress and tumor cell death. Concurrently, the ROS generated by CDT can also induce immunogenic cell death (ICD), releasing tumor-associated antigens (TAAs) and damage-associated molecular patterns (DAMPs).^35,36^ Serving as strong stimuli in the maturation of antigen-presenting cells, these molecules help recruit and activate dendritic cells (DCs).

To boost the targeted delivery of PD-L1 siRNA to hypovascularized lung metastases, a pH-responsive metastatic-targeted catalyst containing tumor-penetrating polymer (TP)/solid lipid-coated Prussian blue (TP-SL@PB) is developed for enhanced PD-L1 siRNA delivery and self-cascade antigen capture to reprogram tumor immunodeficiency (Figure 1a). Through intravenous injection, TP-SL enhances lung metastases penetration by leveraging spontaneous charge conversion and the margination effect (Figure 1b). At the tumor site, the cell–cell interactions were released by the amphiphilic polymer poly­(methoxypoly­(ethylene glycol)-benzoic imine-octadecane, mPEG-*b*-C18) with PB and the nanoparticle-induced endothelial leakage (NanoEL) effect for tumor permeability. This process, combined with penetrating chemodynamic therapy (CDT) and surface charge modulation via Fe^2+^-mediated redox reactions, promotes Fenton-like reactions and endosomal effects. These mechanisms collectively enhance PD-L1 siRNA expression and facilitate the release of tumor-associated antigens (TAAs). Furthermore, the primary amine groups on C18 and the lipids of TP-SL capture TAAs, serving as an antigen reservoir to support immunogenic cell death. The TP-SL@PB system plays a pivotal role in prolonging antigen release and ensuring sustained immune stimulation to inhibit tumor metastasis. Additionally, the captured antigens actively recruit dendritic cells, amplifying the immune response through CD4^+^ and CD8^+^ T cells.

**Figure 1:**
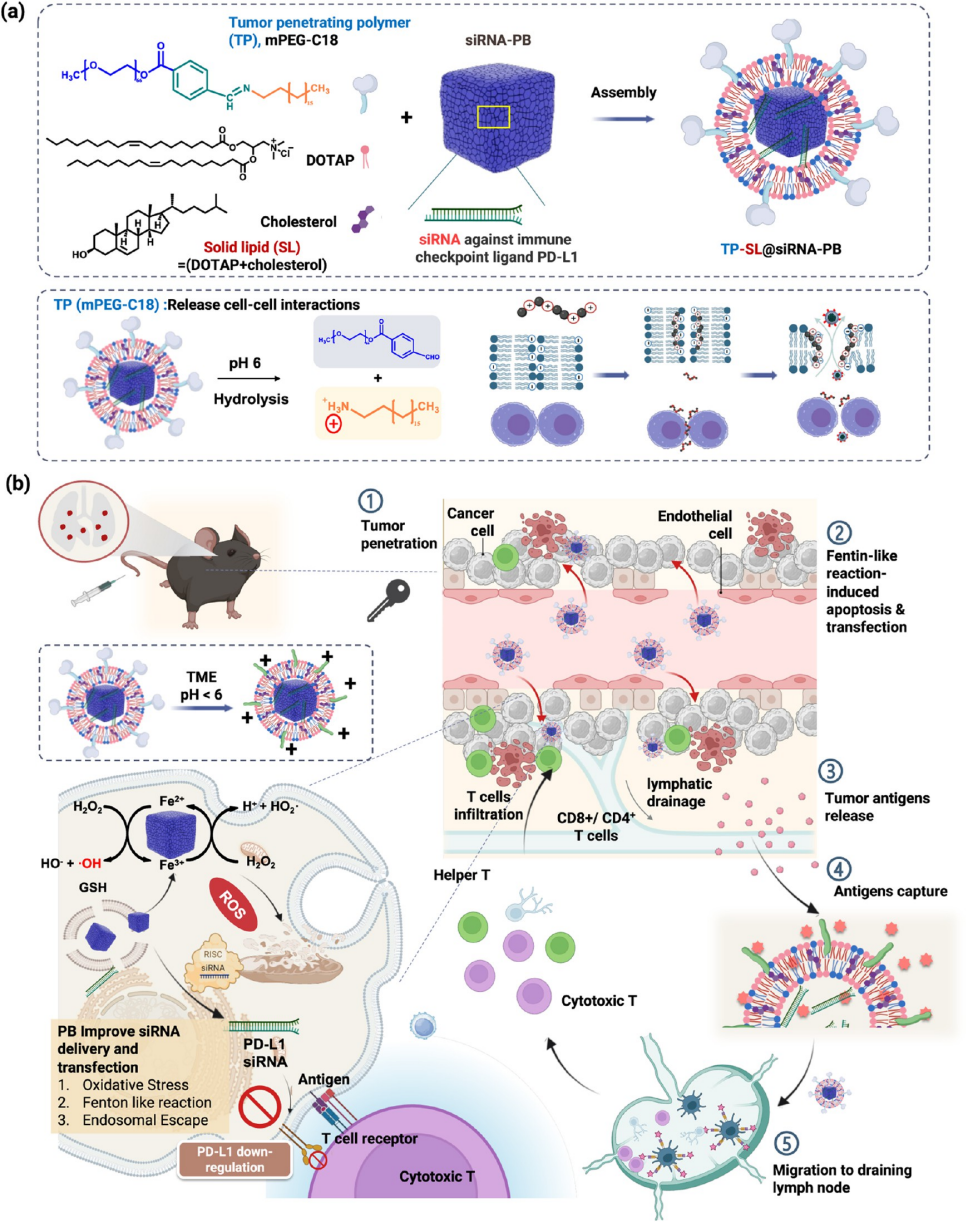
Schematic illustration of a pH-responsive lung metastatic-targeted catalysts containing the tumor penetration polymer (TP)/solid lipids (SL)-coated Prussian blue (TP-SL@PB)-enhanced PD-L1 siRNA delivery and self-cascade antigen capture for reprogramming immunodeficiency. (a) Preparation of TP-SL@PB as a lung metastatic-targeted catalyst. TP as agents by pH-responsive tumor accumulation and released cell–cell interaction; SL as a transfection and organ-selection agents. (b) After intravenous injection, TP-SL@PB accumulated in the blood vessel-poor lung metastases via the organ-selective targeting and charge conversion of TP. The catalytic and tumor-penetrating effects for promoting PD-L1 siRNA delivery and chemodynamic therapy. The induced neoantigens and damage-associated molecular (DAMPs) stimulate the uptake of tumor-associated antigens (TAAs) by phagocytic cells and activate CD4^+^ and CD8^+^ T cells.

## 2 Results and Discussion

### 2.1 Synthesis and Morphology of PB, IO, and TP-SL@IO

The synthesis process of TP-SL@PB composed of positively charged *N*-(2,3-dioleoyloxy-1-propyl) trimethylammonium methyl sulfate (DOTAP, cationic organ-selective lipid and transfection reagent), membrane-disrupted amphiphilic polymer poly­(methoxypoly­(ethylene glycol)-benzoic imine-octadecane, mPEG-*b*-C18, TP) and PB (CDT-boosted margination and transfection) is depicted in Figure 2a. PB was first synthesized via a hydrothermal method using poly­(vinylpyrrolidone) (PVP) and K_3_[Fe­(CN)_6_]. During this process, a crystalline structure formed through the coordination of Fe^2+^ and Fe^3+^ ions with cyanide groups (−CN^–^) from K_3_[Fe­(CN)_6_] in an acidic environment. Subsequently, SL and TP were incorporated into the PB particles through thin-film hydration-assisted self-assembly. Another core system, cubic iron oxide particles (IO), was prepared by calcination at 350 °C for 2 h in an air route. Both PB and IO were approved for clinical use by the FDA.^37^ The byproduct was resuspended in deionized water (D. I. water).

**Figure 2:**
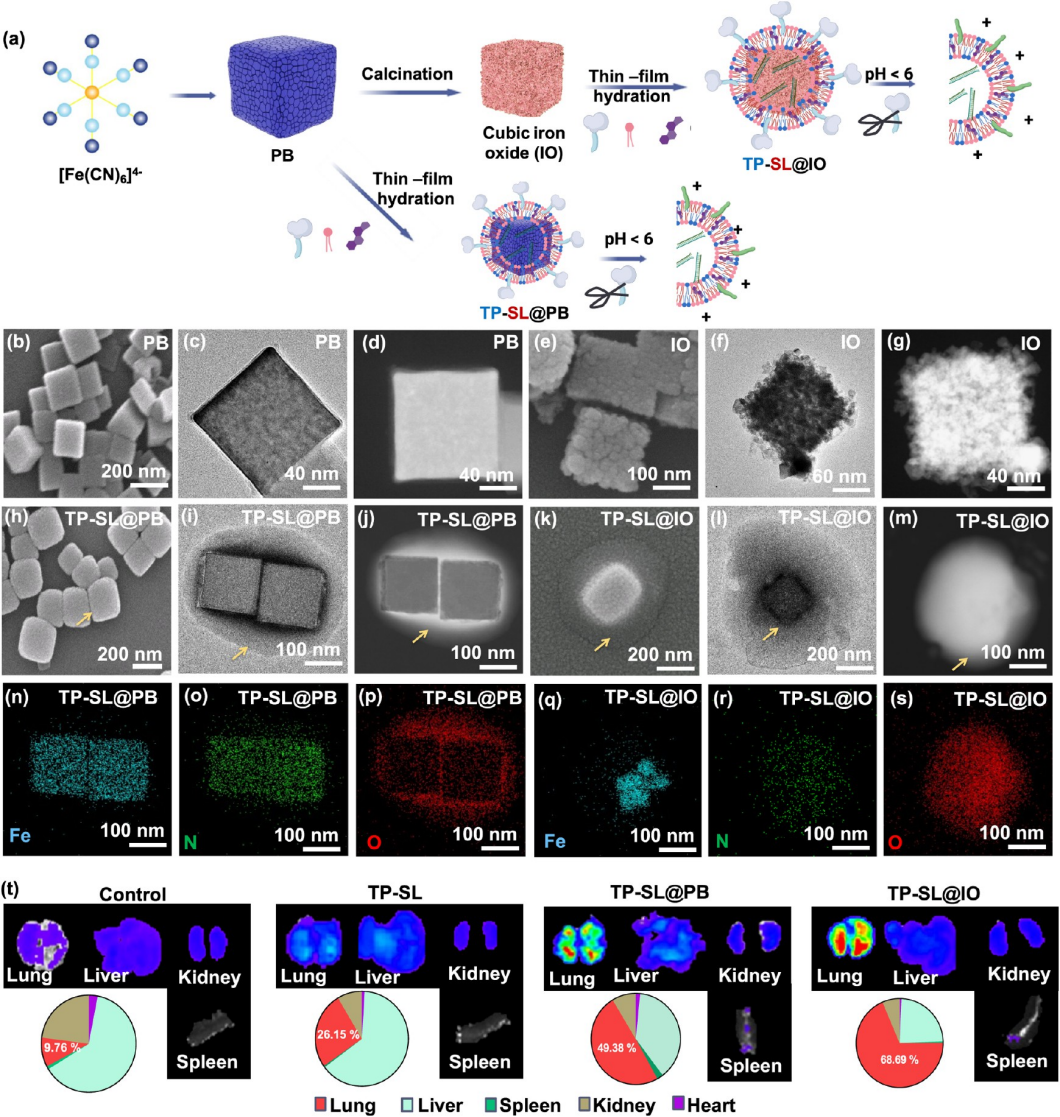
Synthesis and characterization of TP-SL@PB. (a) Schematic illustration of the preparation of TP-SL@PB and TP-SL@IO with siRNA. (b–m) SEM and TEM images of PB, IO, TP-SL@PB, and TP-SL@IO, respectively. (n–s) EDS elemental mapping analysis of TP-SL@PB and TP-SL@IO. (t) *In vivo* IVIS images of the main clearance organs from mice 24 h after treatment with TP-SL, TP-SL@PB, and TP-SL@IO.

The morphologies of PB, IO, and TP-SL@PB were analyzed by using scanning electron microscopy (SEM) and transmission electron microscopy (TEM). As shown in Figure 2b–m, PB, IO, and TP-SL@PB exhibit a cubic structure. PB features a relatively solid structure with smooth, flat edges (Figure 2b–d), while IO displays a rougher surface (Figure 2e–g). The TP-SL coating on the surfaces of PB and IO maintains the cubic morphology, resulting in the formation of core–shell structures (Figure 2h–m). Energy-dispersive X-ray spectroscopy (EDS) spectra (Figure 2n–s) further verify the presence of oxygen species from DOTAP and mPEG-C18, distributed around the periphery of the PB and IO nanoparticles.

### 2.2 Biodistribution of Solid Lipid Particles (LNPs), TP-SL@PB, and TP-SL@IO

To preliminarily evaluate the effects of surface charge and particle composition on biodistribution, lipid-coated nanoparticles with varying components were intravenously administered into mice bearing lung metastatic tumors. Major clearance organs (heart, liver, spleen, lungs, and kidneys) were collected 24 h postinjection and analyzed using *in vivo* imaging system (IVIS) photography. Given the role of helper lipids in biodistribution, a series of lipid-based nanoparticles containing structurally distinct phospholipids—neutral 1,2-dioleoyl-*sn*-glycero-3-phosphocholine (DOPC), anionic 1,2-dimyristoyl-*sn*-glycero-3-phospho-(1’-rac-glycerol) (sodium salt) (DMPG), cationic 1,2-dioleoyl-3-trimethylammonium-propane (DOTAP), and methoxy poly­(ethylene glycol) conjugated to a C18 lipid chain (mPEG-C18) (lipid:mPEG-C18:cholesterol = 5:1:4)—were screened (Figure S1). Results indicated that cationic DOTAP particles achieved the highest lung accumulation, consistent with literature documenting DOTAP’s positive charge enhancing electrostatic interactions with negatively charged lung surfaces, promoting retention and accumulation (Figure S2).^30^

Increasing the ratio of mPEG-C18 in DOTAP formulations enhances lung accumulation efficacy (Figure S3). This effect is attributed to improved nanoparticle stability and reduced clearance by the mononuclear phagocyte system. PEGylation provided by mPEG-C18 forms a hydrophilic steric barrier around the nanoparticles, minimizing nonspecific interactions with serum proteins and immune cells and thus prolonging the circulation time. The increased stability allows more DOTAP-based nanoparticles to reach the lungs, where their positive charge interacts effectively with negatively charged pulmonary tissues, enhancing the accumulation efficiency.

After the effects of lipids and polymers were evaluated, IO and PB were incorporated into the lipid/polymer system to form TP-SL@PB and TP-SL@IO particles, respectively, for distribution studies. IVIS imaging (Figure 2t) shows that lung accumulation increased to approximately 50 and 69% for TP-SL@PB and TP-SL@IO particles, respectively. This enhanced accumulation is attributed to the phenomenon of marginated delivery in the blood, where particles preferentially accumulate in the lungs due to vascular dynamics. This mechanism facilitates passive targeting of lung metastases by exploiting vascular leakage, enabling effective delivery of therapeutic agents to metastatic sites.^38–42^ For both the PB and IO groups, particle accumulation was predominantly observed in the liver and spleen (Figure S4). Such systems hold promise for advancing therapies aimed at pulmonary diseases and metastases through targeted and efficient delivery mechanisms.

### 2.3 Characterization of PB and TP-SL@PB

The effects of surface modification by SL and TP on the hydrodynamic size and ζ-potential of the particles were analyzed. Dynamic light scattering (DLS) measurements revealed that the initial diameters of PB and IO nanoparticles were approximately 210 and 230 nm, respectively (Figure 3a,b). Following surface coating with TP and SL, the hydrodynamic sizes increased to 237 nm for TP-SL@PB and 261 nm for TP-SL@IO. This increase in size indicates successful deposition of the lipid layers and TP molecules on the nanoparticle surfaces. Further, the stability of TP-SL@PB in aqueous medium was evaluated by the hydrodynamic diameter, polydispersity index (PDI) of nanoparticles incubated in distilled water or phosphate-buffered saline (PBS) for different times. As shown in Figure S5, the average hydrated dynamic particle size of TP-SL@PB determined by DLS did not change significantly and PDI remained lower than 0.25 upon incubation in DI water or PBS for 7 days, revealing that TP-SL@PB has excellent dispersibility and stability in aqueous medium. In addition, the modification with cationic DOTAP lipids significantly altered the surface charge of the nanoparticles. Initially, the ζ-potentials of PB and IO were measured at −23.28 and −33.31 mV, respectively. After coating with DOTAP lipids, the ζ-potentials shifted to strongly positive values of 28.3 mV for TP-SL@PB and 27.5 mV for TP-SL@IO (Figure 3c). This shift demonstrates the electrostatic effect of the DOTAP lipids, which not only stabilize the nanoparticles in suspension but also enhance their potential for interaction with negatively charged biological membranes.

**Figure 3:**
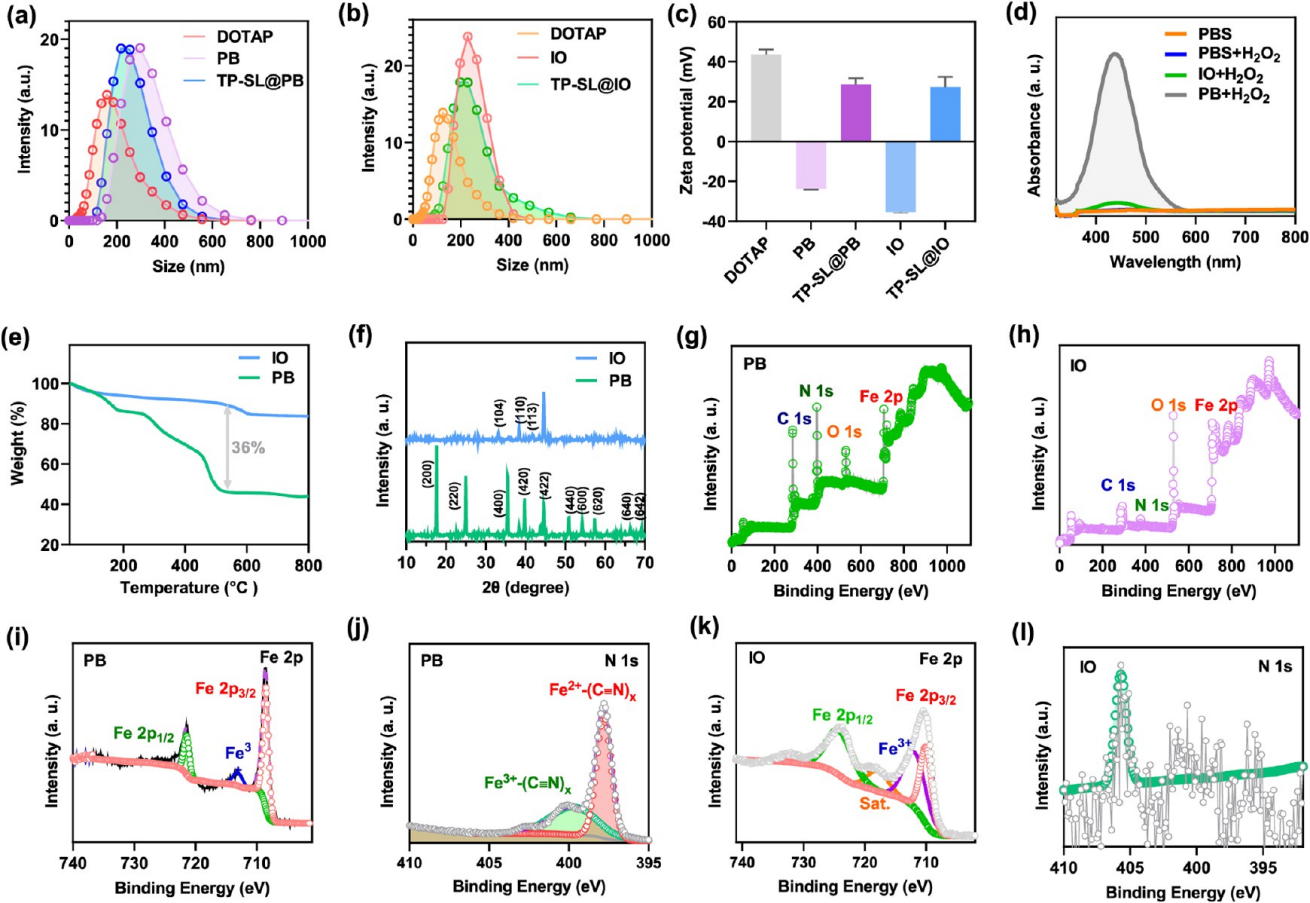
Physicochemical characterization of TP-SL@PB and TP-SL@IO. (a, b) Size distribution and (c) ζ-potential of PB, DOTAP LNP, TP-SL@PB, and TP-SL@IO. (d) UV–vis adsorption spectra of catalyzed oxidation of *o*-phenylenediamine (OPD) after treating with PBS (control), PB, and IO. (e) Thermogravimetric analysis (TGA) of PB and IO. (f) X-ray diffraction patterns of PB and IO. (g, h) XPS spectra of PB and IO. (i–l) High-resolution XPS spectra of Fe 2p and N 1s of PB and IO.

The catalytic performance of PB was assessed based on their ability to generate reactive oxygen species (ROS). The oxidation of o-phenylenediamine (OPD) was used as an indicator with the absorbance of oxidized OPD measured to quantify ROS production. In Figure 3d, both PB and IO nanoparticles catalyzed the oxidation of OPD, and PB exhibited significantly higher ROS generation compared to IO nanoparticles (Figure S6). This superior catalytic performance of PB can be attributed to its robust crystalline structure and the efficient redox cycling between Fe^2+^ and Fe^3+^ ions, which facilitate electron transfer and enhance catalytic activity. The enhanced ROS production by PB nanoparticles underscores their potential for applications in oxidative stress-mediated therapies, such as CDT. Furthermore, thermogravimetric analysis (TGA) was performed to confirm the calcination efficacy on the surface of PB (Figure 3e).

The X-ray diffraction (XRD) spectra revealed that both PB and IO nanoparticles possess a face-centered-cubic lattice structure (Figure 3f), with well-defined diffraction peaks corresponding to the Miller indices ((200), (220), (222), (400), (420), and (422)).(40) These peaks confirm the high crystallinity of the synthesized nanoparticles. The X-ray photoelectron spectroscopy (XPS) analysis (Figure 3g–l) provided further insights into the elemental composition and bonding states of the PB and IO nanoparticles. The binding energies corresponding to C 1s, N 1s, O 1s, and Fe 2p were observed, indicating the successful synthesis of the nanoparticles. For PB nanoparticles, the high-resolution C 1s spectra revealed peaks at 284.8, 285.6, 286.5, and 288.3 eV, corresponding to C–C, C≡N, C = O, and O–C = O bonds, respectively. The N 1s spectra showed two prominent peaks: one at 397.8 eV, attributed to Fe^2+^–(C≡N)_*x*_, and another at 399.8 eV, corresponding to Fe^3+^–(C≡N)_*x*_. In the case of IO nanoparticles, distinct peaks were observed in the Fe 2p region, including Fe 2p_3_/_2_ at 708.5 eV, Fe^3+^ at 713.2 eV, and Fe 2p_1_/_2_ at 721.5 eV. These results validate the incorporation of iron within the IO structure and align with its expected oxidation states.

### 2.4 Cell Uptake and Cytotoxicity of TP-SL@PB

To investigate the intracellular uptake and distribution of particles in cells, TP-SL, TP-SL@PB, and TP-SL@IO were cocultured with B16F10 cells (a murine tumor cell line; skin melanoma cells) under varying concentrations and durations. For these experiments, the particles were fluorescently labeled with Cy5-siRNA (red fluorescence) to enable the tracking of siRNA delivery. After 8 h of incubation, the nuclei and cytoskeleton of B16F10 cells were stained with DAPI (blue fluorescence) and F-actin (green fluorescence), respectively, and the cells were imaged using confocal laser scanning microscopy (CLSM). In Figure 4a, only a limited number of TP-SL adhered to the cell membrane. In contrast, TP-SL@PB and TP-SL@IO nanoparticles exhibited greater accumulation in the cytoplasm after 8 h of coculture, with some particles even surrounding the cell nucleus. The quantification of fluorescence intensities in cells showed similar results (Figure 4b).

**Figure 4:**
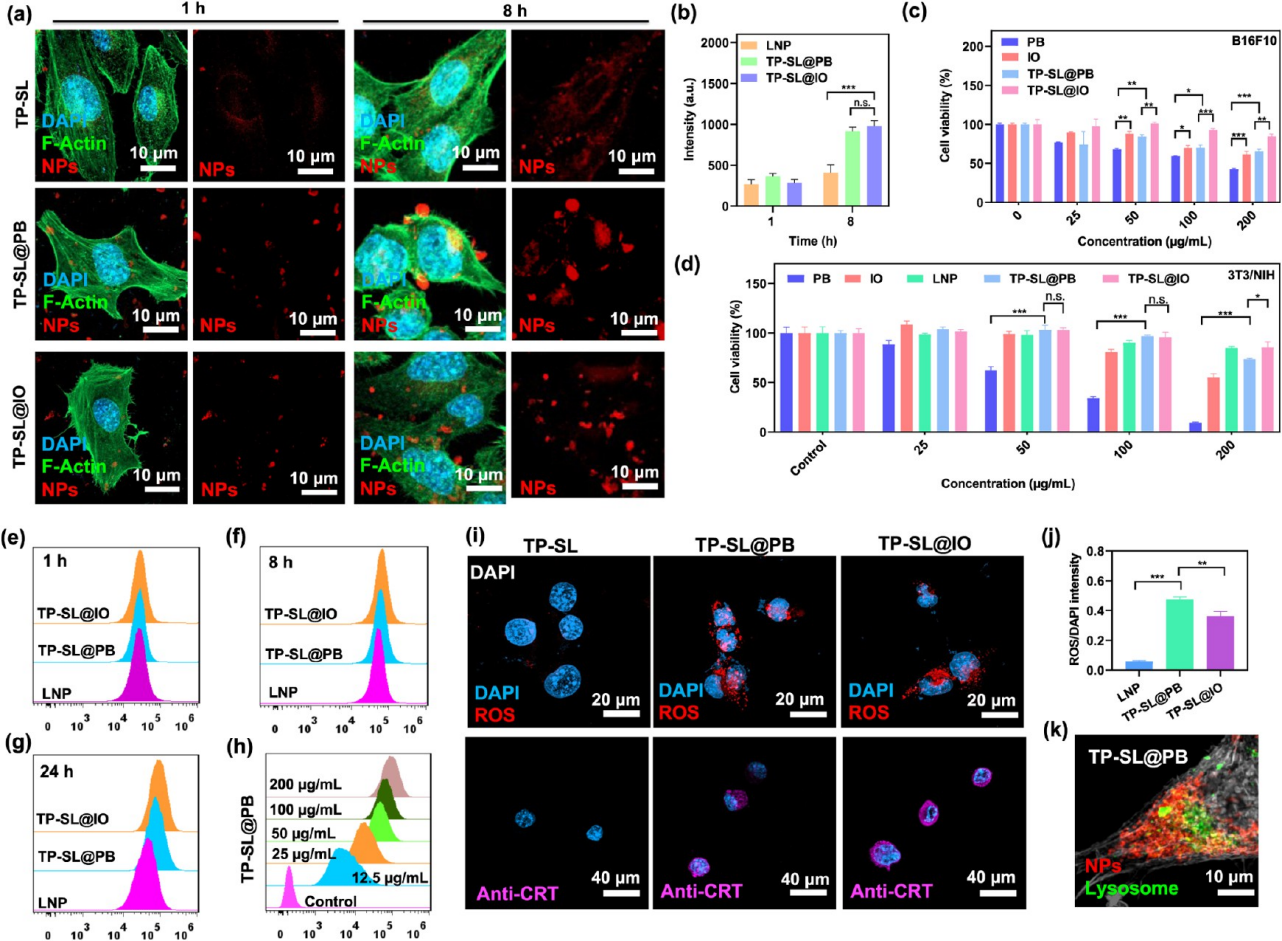
*In vitro* studies and ROS generation of TP-SL@PB. (a) CLSM images of B16F10 cells after incubation with particles for 8 h and (b) quantification of cellular uptake. Blue, green, and red displayed nucleus stained with DAPI, cytoskeleton with F-Actin, and NPs stained with cy5-siRNA, respectively. Error bars represent mean ± SEM, *n* = 6; ***p < 0.001 compared with the group by one-way ANOVA with Tukey’s multiple comparison test. The cytotoxicity after 24 h of incubation with (c) B16F10 and (d) 3T3NIH cell lines. Error bars represent mean ± SEM, *n* = 6; *p < 0.5, **p < 0.01, and ***p < 0.001 compared with the group by one-way ANOVA with Tukey’s multiple comparison test. Flow cytometry analysis of TP-SL, TP-SL@PB, and TP-SL@IO in the B16F10 cell line after incubation for (e) 1 h, (f) 8 h, and (g) 24 h. (h) Flow cytometry analysis of TP-SL@IO in B16F10 cell line after incubation at various concentrations. (i) CLSM images and (j) quantification of ROS generation of the B16F10 cell line after treatment with TP-SL, TP-SL@PB, and TP-SL@IO. Error bars represent mean ± SEM, *n* = 5; **p < 0.01, and ***p < 0.001 compared with the group by one-way ANOVA with Tukey’s multiple comparison test. All data are presented as the mean ± SEM. One-way ANOVA with Tukey’s multiple comparison test and an unpaired two-tailed *t* test was used to assess statistical significance *P* values, which are NS, not significant; *P < 0.05, **P < 0.01, ***P < 0.001, ****P < 0.0001. (k) CLSM images of mitochondria of B16F10 cells after treatment with TP-SL@PB.

The cytotoxicity of the particles was evaluated by incubating them with B16F10 cells and assessing the cell viability over time. An increase in particles concentration increased in cytotoxicity in the PB and TP-SL@PB groups, attributed to their stronger ROS generation effects (Figure 4c). In contrast, the TP-SL@IO group exhibited a lower cytotoxicity. When the nanoparticle concentration was increased to 100 μg/mL, PB alone demonstrated the ability to kill approximately 40% of the B16F10 cells (Figure 4c).

In addition, NIH/3T3 cells (mouse embryonic fibroblasts) were used to further evaluate the cell viability. Results showed low cytotoxicity in NIH/3T3 cells across all nanoparticle groups (Figure 4d). This reduced toxicity may be attributed to the lower ROS generation in these cells, which could stem from differences in the cellular uptake or oxidative stress tolerance between fibroblasts and cancer cells. These findings highlight the selective cytotoxicity of PB and TP-SL@PB, making them promising candidates for targeted cancer therapy. Meanwhile, the reduced toxicity of TP-SL@IO and the biocompatibility with NIH/3T3 cells suggest their potential for safer therapeutic applications or use in noncancerous environments.

### 2.5 Flow Cytometry of TP-SL@PB

Flow cytometry analysis was conducted to investigate the time-dependent cellular uptake of the TP-SL, TP-SL@PB, and TP-SL@IO nanoparticles. Figure 4e–g illustrates the uptake profiles over time, from 1 to 24 h. At the first hour, no significant differences were observed among the three groups in terms of cell uptake, suggesting similar initial interactions with the cell membrane. However, by the 24th hour, both TP-SL@PB and TP-SL@IO demonstrated significantly higher cellular uptake efficiency compared to TP-SL alone. This enhanced uptake is likely attributable to the PB and IO cores, which may promote more robust nanoparticle internalization through mechanisms, such as receptor-mediated endocytosis.

In the concentration-dependent study, increasing the dose of TP-SL@PB led to a marked enhancement in the uptake efficiency (Figure 4h). This dose–response relationship highlights the potential to optimize therapeutic delivery by modulating nanoparticle concentration. Nanoparticles, with their distinct physicochemical properties, may facilitate cellular uptake by disrupting the cell membrane or by engaging specific pathways, such as clathrin- or caveolae-mediated endocytosis. Moreover, the lipid coating likely provides additional stabilization and enhances compatibility with the cell membrane, further contributing to the observed efficiency.

### 2.6 ROS Generation *In Vitro* of TP-SL@PB

The ability of TP-SL, TP-SL@PB, and TP-SL@IO to generate reactive oxygen species (ROS) was assessed through a Fenton reaction using the Mitochondrial Hydroxy Radical Detection Assay Kit. Nuclei were stained with Hoechst 33342 (blue), and ROS generation was examined using CLSM after the addition of the OH580 working solution, a ROS-specific stain. Each formulation was added at a concentration of 100 μg/mL to trigger ROS production through the Fenton reaction in the B16F10 cells. In Figure 4i,j, TP-SL@PB and TP-SL@IO produced significantly more ROS compared to the TP-SL. This increased ROS generation can be attributed to the catalytic activity of PB and IO nanoparticles, which, in the presence of hydrogen peroxide (H_2_O_2_) in B16F10 cells, produce hydroxyl radicals (OH^•^) through the Fenton reaction. These reactive species contribute to oxidative stress, which can damage cellular components, ultimately leading to cell death.

Calreticulin (CRT) is a crucial marker of immunogenic cell death (ICD), a process initiated by the translocation of CRT from the endoplasmic reticulum (ER) lumen to the nuclear surface. Once exposed, CRT serves as an “eat-me” signal, facilitating recognition by CD91-expressing antigen-presenting cells (APCs) such as macrophages and dendritic cells (DCs). This interaction enhances DC recruitment and promotes antigen presentation. In Figure 4i, CRT (purple fluorescence) was detected in TP-SL@PB and TP-SL@IO-treated cells. High Mobility Group Box 1 (HMGB1) also plays a key role in the immune response as a pro-inflammatory mediator. Under stress, injury, or necrosis, HMGB1 is either actively secreted by immune cells or passively released from damaged cells. Once extracellular, it acts as a damage-associated molecular pattern (DAMP), alerting the immune system to tissue damage or infection. Cancer cells treated with TP-SL@PB exhibited strong HMGB1 expression (Figure S7), indicating its involvement in the immune-stimulatory effects of the treatment. This phenomenon may be attributed to stress-induced upregulation of HMGB1 expression at the transcriptional level prior to its extracellular release. Additionally, TP-SL@PB might also alter the kinetics of HMGB1 translocation by modulating the nuclear membrane integrity and intracellular redox signaling, leading to transient nuclear retention. Previous research has indicated that the multiple functions of extracellular HMGB1 might be attributed to its different redox states in a context-dependent manner. HMGB1 can also be released by cells undergoing secondary necrosis and by cancer cells during ICD.^43^

The enhanced ROS generation by TP-SL@PB and TP-SL@IO suggests that these nanoparticles can induce a potent cytotoxic effect. In the context of gene delivery, ROS generation can be strategically used to enhance the release and efficacy of therapeutic siRNA or DNA. The oxidative stress environment generated by PB and IO may improve the permeability of cellular membranes or endosomal escape, facilitating the release of nucleic acids from the nanoparticles. Furthermore, the induced cell death may amplify the therapeutic effect of the gene delivery systems. The positive charge of TP-SL@PB contributes to its ability to escape from mitochondria and lysosomes through the proton sponge effect (Figures 4k, S8 and S9). This phenomenon occurs when the amine-rich TP-SL@PB absorbs protons within the acidic lysosomal environment, leading to osmotic swelling and the eventual rupture of the lysosomal membrane.

To preliminarily assess the effects of TP-SL@PB and TP-SL@IO on dendritic cell (DC) maturation, B16F10 cells were treated with TP-SL@PB and TP-SL@IO, and their released antigens were collected. These antigens, combined with TP-SL@PB, were then cocultured with DC2.4 cells—a murine dendritic cell line commonly used in DC biology research, for 24 h. After the coculture, DC2.4 cells were fixed and stained for iNOS and CD80, markers that distinguish immature from mature DCs. In Figure S10, CLSM images revealed that DC2.4 cells treated with antigens@TP-SL@PB exhibited signs of maturation, indicating that antigen-containing particles can promote DC maturation.

### 2.7 Cell Membrane Leakage of TP-SL@PB

To assess molecule-induced cell membrane leakage, a method adapted from Kuroda et al. was employed.^44^ In this approach, DAPI was used as a marker to evaluate the membrane integrity; in healthy cells, the membrane prevents DAPI entry, serving as a barrier. However, when the membrane is compromised, DAPI can enter rapidly and label the nucleus. To explore the enhanced membrane permeability induced by various particles, fluorescence changes of DAPI were monitored after 30 min of incubation using CLSM (Figure 5a). In the control group, weak DAPI fluorescence was observed, consistent with prior findings that DAPI penetrates intact membranes inefficiently at low concentrations (1 μg/mL). Both TP-SL and TP-SL@PB exhibited significant cell membrane leakage, suggesting that these particles effectively induce membrane disruption compared with the control group.

**Figure 5:**
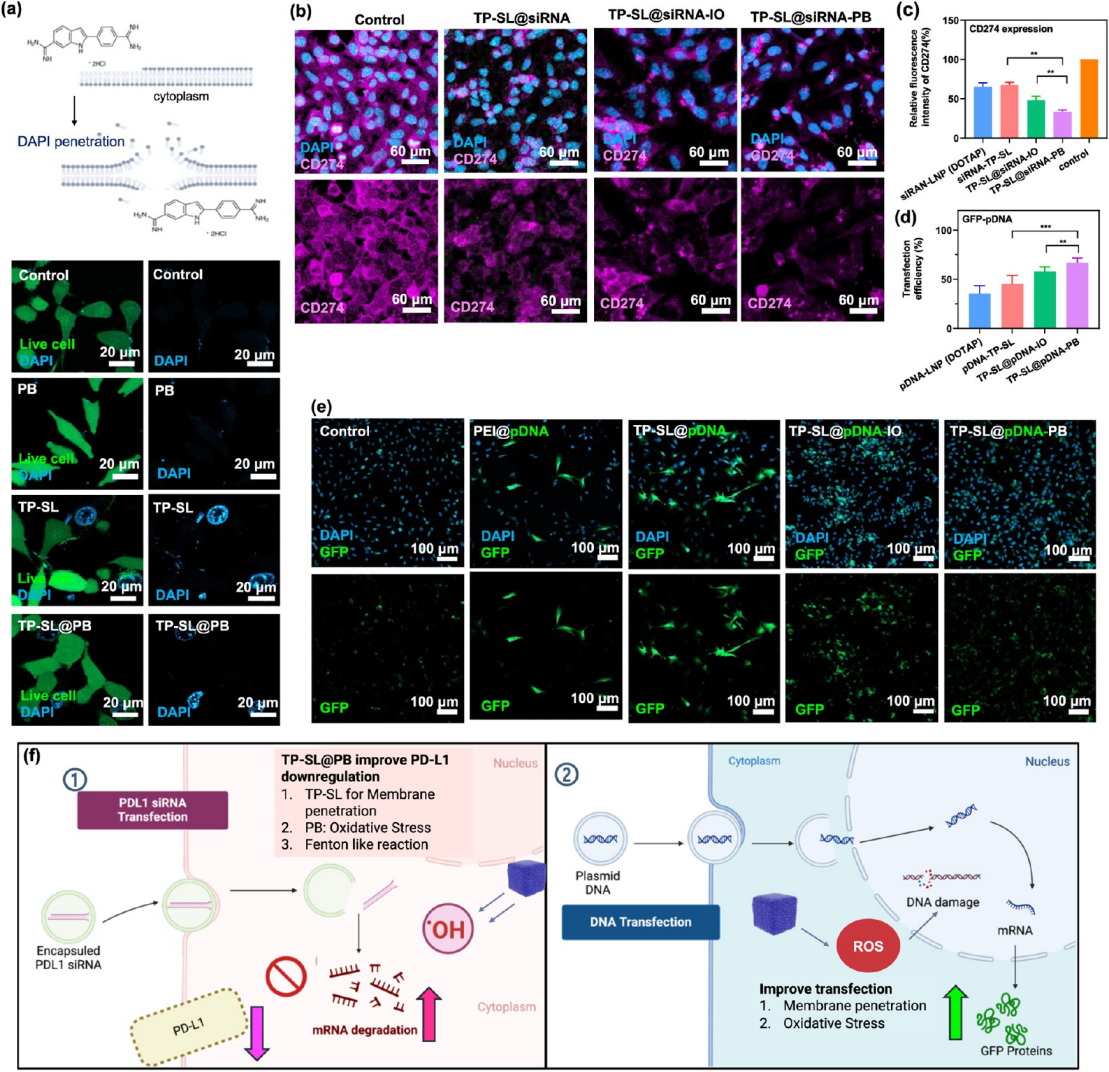
(a) Membrane disruption in TP-SL and TP-SL@PB evaluated using DAPI staining visualization and LIVE cytotoxicity kit to assess membrane disruption indicators and cell morphology, respectively. (b) CLMS images of the expression of PD-L1 siRNA-mediated protein knockdown, CD247, after 24 h coculturing with TP-SL, TP-SL@PB, and TP-SL@IO. Quantification of expression of (c) CD247 and (d) GFP-pDNA after various treatments. Error bars represent mean ± SEM, *n* = 3; **p < 0.01, and ***p < 0.001 compared with the group by one-way ANOVA with Tukey’s multiple comparison test. (e) CLSM images of GFP expression after treatment with GFP-pDNA-loaded with particles incubated with B16F10 cells. (f) The scheme of the mechanism of TP-SL@PB for enhancing PD-L1 siRNA and GFP-pDNA transfection.

To visualize the interaction between cells and TP-SL, SEM was performed after an 8 h incubation with 50 μg/mL TP-SL and TP-SL@PB (Figure S11). SEM images revealed that cells treated with TP-SL@PB exhibited changes in surface morphology, including increased membrane folding and roughness. These alterations suggest that the positively charged TP-SL@PB nanoparticles interact strongly with the negatively charged cell membrane, leading to membrane disruption and structural deformation. The cationic nature of TP-SL@PB facilitates electrostatic attraction to the anionic components of the cell membrane, such as phospholipids and glycoproteins. This interaction can destabilize the membrane integrity, causing physical stress, increased permeability, and morphological changes.

### 2.8 PD-L1 siRNA-Mediated Protein Knockdown by TP-SL@PB

After the membrane-penetration effects were demonstrated, the delivery efficacy of PD-L1 siRNA via TP-SL@PB was further assessed. EDS mapping images of TP-SL@siRNA-PB displayed the distribution of phosphorus (P) elements, revealing the successful integration of siRNA into TP-SL@PB (Figure S12a). The PD-L1 knockdown efficiency and its effects on B16F10 cells were evaluated using PD-L1 siRNA-loaded TP-SL and TP-SL@PB at an siRNA concentration of 1 μg/mL in serum-free medium. The siRNA loading efficiency (EE%) in TP-SL@PB was approximately 95.6%. The cumulative siRNA release profile is presented in Figure S12b. TP-SL@PB exhibited a strong affinity for siRNA due to robust electrostatic interactions, leading to a slow sustained release behavior. The evaluation involved examining sequence-specific knockdown of PD-L1 mRNA, mediated by the targeted siRNA. Programmed cell death 1 ligand 1 (PD-L1), also known as cluster of differentiation 274 (CD274) or B7 homologue 1 (B7–H1), is a protein encoded by the CD274 gene in humans. Consequently, CD274 expression serves as a crucial indicator for assessing the delivery of PD-L1-specific siRNA.

CLSM images in Figure 5b illustrate the outcomes of PD-L1 siRNA delivery. PD-L1 siRNA-loaded TP-SL@PB demonstrated a marked reduction in PD-L1 expression compared with the TP-SL group, as assessed by immunohistochemical staining and flow cytometry analysis (Figure 5c). Specifically, relative to the control group, the TP-SL formulation reduced PD-L1 expression by approximately 62%. In contrast, the expression of PD-L1 in the TP-SL@IO and TP-SL@PB groups was significantly lower, with reductions of 50.2 and 41%, respectively. In addition, the PD-L1 expression in the B16F10 cell line was confirmed by Western blot analysis, and the results showed a lower expression pattern in the TP-SL@siRNA-PB group (Figure S13). These results suggest that the Fenton reaction induced by PB enhances the efficacy of PD-L1 siRNA, likely through the generation of reactive oxygen species (ROS) that contribute to an enhanced therapeutic response.

These formulations were also evaluated for *in vitro* gene delivery using EGFP plasmid DNA (pDNA) to assess their efficiency in driving gene expression. Consistent with the siRNA delivery results, B16F10 cells treated with TP-SL@PB and TP-SL@IO exhibited significantly higher levels of GFP expression compared to the other groups (Figure 5d,e). This enhanced gene expression can be attributed to the superior delivery efficiency and the synergistic effects facilitated by the TP-SL@PB and TP-SL@IO formulations. These findings further underscore the potential of these nanoplatforms to serve as effective vectors for both siRNA and pDNA-based gene delivery applications.^41^

The observed decreases in PD-L1 expression indicate the efficacy of the siRNA delivery system and its ability to modulate tumor immunosuppression (Figure 5f). The superior performance of TP-SL@PB over TP-SL can be attributed to the presence of PB, which facilitates the Fenton reaction, producing ROS that may act synergistically with PD-L1 siRNA to disrupt tumor immune evasion mechanisms. The significant reduction observed in the TP-SL@PB group highlights the potential for membrane penetration and combining gene silencing strategies with catalytic therapies to achieve enhanced therapeutic outcomes.

### 2.9 Margination and Penetration of Particles in Tumor Spheroids

Three-dimensional (3D) *in vitro* tumor models provide a more accurate representation of the architecture and microenvironment of solid tumors, offering improved predictions of clinical outcomes. To investigate the penetration capabilities, multicellular tumor spheroids (MTSs) composed of B16F10 cells and NIH/3T3 cells were generated using a microfluidic chip array (Figure 6a).^45^ This approach successfully produced an array of MTSs with a high formation rate (>98%) and remarkable uniformity, achieving an average tumor size of approximately 200 μm in diameter (Figure 6b–d). The confinement of microdroplets facilitated the formation of tight cell-to-cell connections.

**Figure 6:**
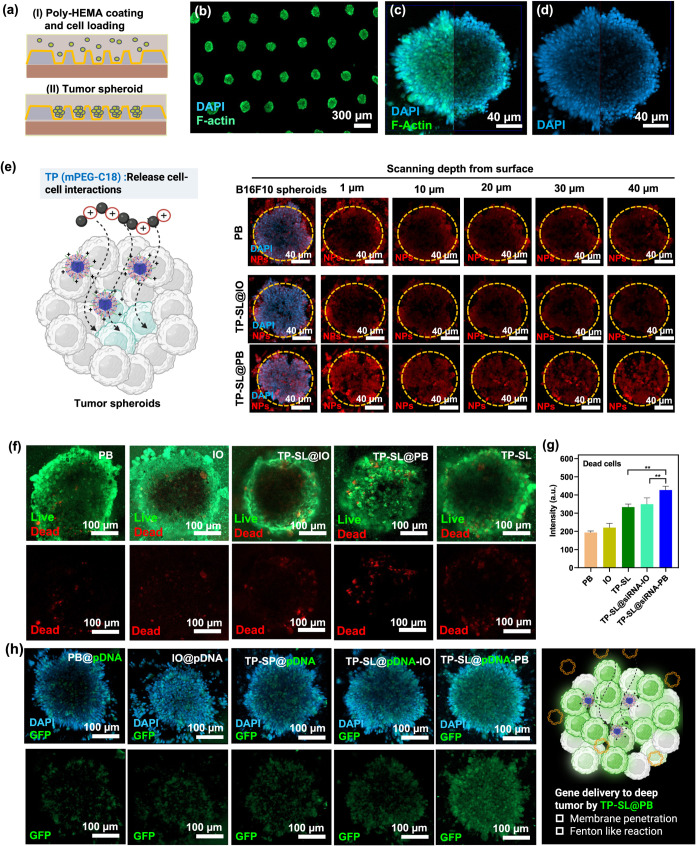
(a) Schematic illustration of multicellular tumor spheroid (MTS) preparation on a microfluidic chip through coculturing B16F10 and NIH/3T3 cells. (b–d) CLSM images of untreated MTSs, with nuclei in blue and F-actin in green. (e) CLSM images of MTSs treated with PB, TP-SL@IO, and TP-SL@PB, with Cy5-siRNA-labeled particles shown in red fluorescence. (f) Live/dead images and (g) qualification of fluorescence intensity of MTS after treatment with PB, IO, TP-SL@PB, TP-SL@IO, and TP-SL@PB. Error bars represent mean ± SEM, *n* = 6; **p < 0.01 compared with the group by one-way ANOVA with Tukey’s multiple comparison test. (h) CLSM images of GFP-pDNA expression in MTS after treatment with various particles.

To evaluate the particle penetration effects, the particles were labeled with a red fluorescent dye, 1,1’-dioctadecyl-3,3,3′,3′-tetramethylindocarbocyanine Perchlorate,^30^ which has an absorption maximum at 549 nm and an emission maximum at 565 nm. After 1 day of incubating the multicellular tumor spheroids (MTSs) with these particles, depth profiles were scanned using CLSM to assess penetration efficiency. CLSM images in Figure 6e, TP-SL@PB and TP-SL@IO exhibited significantly better penetration depths within the MTSs, indicating that the TP-SL formulation enhances particle penetration. The enhanced penetration can be attributed to the positively charged polymers within the formulation, which disrupt cell membranes through electrostatic interactions with the negatively charged phospholipids in the membrane. These interactions destabilize the membrane, allowing the polymer to insert into the lipid bilayer, thereby forming pores or holes that increase membrane permeability.^46,47^ Additionally, the accumulation of positive charges induces mechanical stress, further compromising membrane integrity.^48,49^ This dual mechanism likely contributes to the observed improvement in the penetration depth and efficiency.

In Figure 6f,g, live/dead cell imaging provides more insight into the effects of different treatments on cell viability. When treated with PB or IO alone, only a limited number of dead cells were observed in the central region of MTSs. In contrast, treatment with TP-SL@PB resulted in a significant increase in the number of dead cells in the central region, highlighting the enhanced penetration ability of the particles and the effectiveness of the CDT effect.

### 2.10 Penetrated Gene Delivery in Tumor Spheroids

Following the evaluation of tumor penetration efficiency, EGFP plasmid DNA (pDNA) delivery by the particles was also investigated in the MTSs. At 2 days postculture, MTSs were treated with pDNA-loaded IO, PB, TP-SL, TP-SL@IO, and TP-SL@PB at a concentration of 1 μg/mL in serum-free medium. Gene expression was monitored using CLSM, where green fluorescent protein (GFP) indicated pDNA expression, and the nuclei were stained with DAPI. The results revealed a low GFP intensity and limited distribution primarily near the surface of the spheroids for the PB group (Figure 6h). In contrast, the TP-SL@PB group exhibited significantly stronger GFP signals that penetrated deeper into the inner regions of the MTSs. This finding highlights the superior ability of the TP-SL@PB formulation to enhance pDNA delivery within the spheroid structure. Together, these data indicate that the TP-SL and TP-SL@PB groups significantly improve targeting, penetration, and gene expression *in vitro* compared to those of other formulations.

These findings underscore the importance of particle design in achieving efficient gene delivery in 3D tumor models. The low GFP expression observed in the PB may be attributed to the limited penetration of the particles into the dense spheroid architecture, resulting in restricted pDNA delivery to the outermost cells. Conversely, the enhanced GFP expression and deeper penetration seen with TP-SL@PB suggest that the Fenton reaction-mediated effects and the positive surface charge of the TP-SL@PB particles improved both cellular uptake and intercellular transport within the spheroids. The enhanced gene delivery efficiency of TP-SL@PB is likely due to the combined benefits of the chemodynamic effects provided by PB and the optimized surface properties of TP-SL. Specifically, the CDT induced by PB generates ROS, which may facilitate membrane destabilization and improve intracellular delivery.

### 2.11 *In Vivo* Lung Metastasis Penetration and PD-L1 siRNA Delivery by TP-SL@PB

To evaluate the penetration of TP-SL-based particles in metastatic tumors *in vivo*, GFP-B16F10 cells were implanted in C57B6/L mice to establish a metastatic lung model. After 14 days, different formulations were administered. At 24 h postinjection, the lungs were harvested and sectioned to examine the distribution of particles. In Figure 7a, CLSM images revealed stronger fluorescence signals in tumor regions for TP-SL@IO and TP-SL@PB compared to controls and TP-SL. Minimal fluorescence in normal cell regions indicates the superior penetration and retention ability of TP-SL@IO and TP-SL@PB. These findings align with *in vitro* MTS studies. Flow cytometry further confirmed excellent colocalization of GFP-B16F10 cells and particles *in vivo* (Figure 7b,c). This enhanced penetration and retention can be attributed to the nanoparticles’ ability to exploit the abnormal vasculature and enhanced permeability of metastatic tumors, leading to selective accumulation.

**Figure 7:**
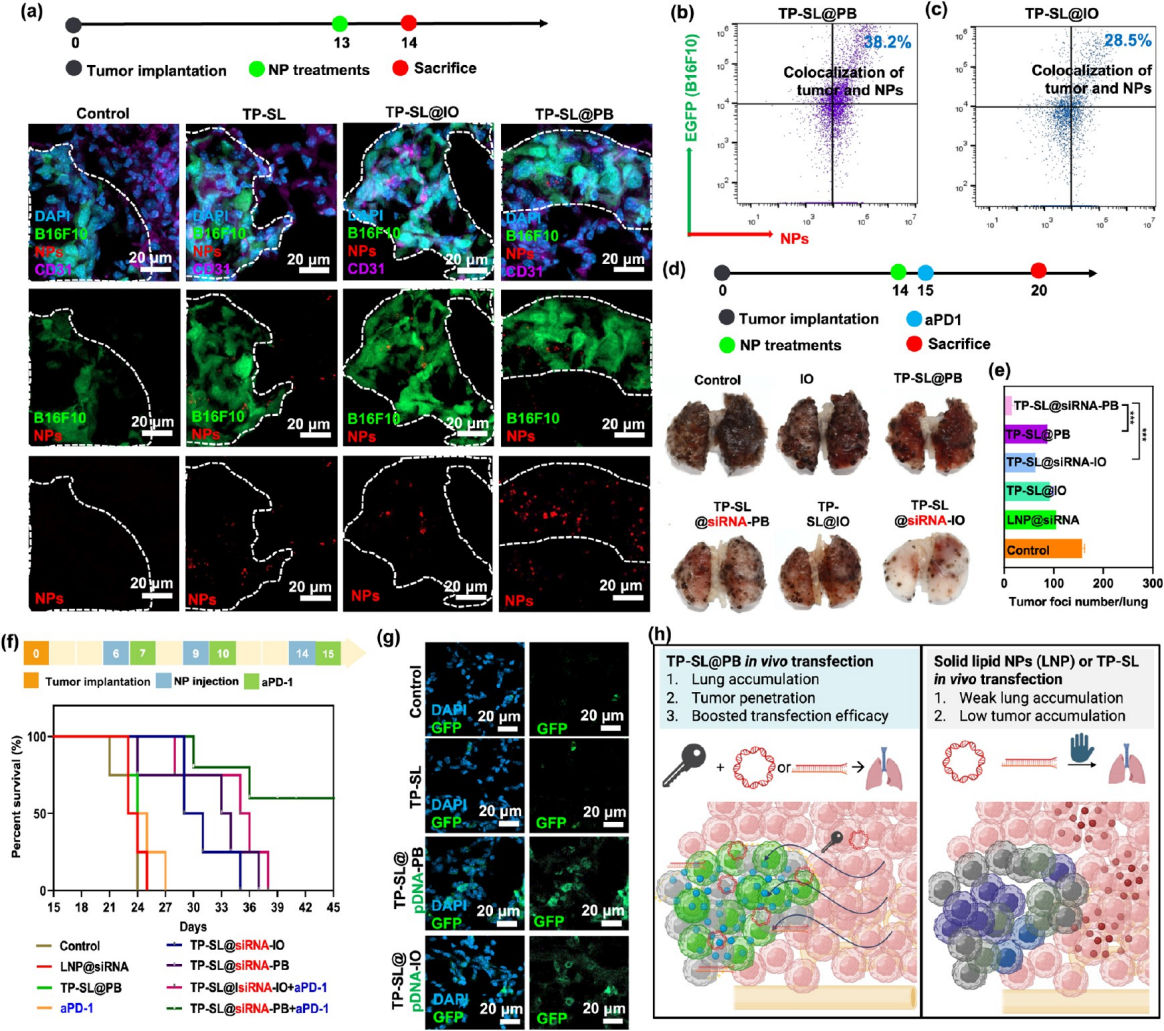
(a) CLSM images of GFP-B16F10 lung metastases administered with cy5-siRNA-labeled TP-SL, TP-SL@PB, and TP-SL@IO at 24 h after injection. (b, c) Representative quantification of colocalization between B16F10 lung metastases and cy5-siRNA-labeled TP-SL@PB, and TP-SL@IO by flow cytometry analysis. (d) Lung tumor suppression images treated with PBS (control), IO, TP-SL@PB, TP-SL@siRNA-PB, TP-SL@IO, and TP-SL@siRNA-IO. (e) Tumor foci numbers after various treatments. Error bars represent mean ± SEM, *n* = 6; ***p < 0.001 compared with the group by one-way ANOVA with Tukey’s multiple comparison test. (f) Survival curves of animals with lung metastases following different treatments (*n* = 6). (g) CLSM images of GFP expression in lung metastases following different treatments. (h) The mechanism of TP-SL@PB for enhancing gene delivery *in vivo*.

To investigate the impact of nanoparticles and siRNA on antitumor immunity, mice were treated on days 6 and 9, and then sacrificed on day 14. The lungs were harvested, and the number of metastatic foci in each mouse was quantified. For therapeutic evaluation, PD-L1 siRNA targeting the nucleus was selected to assess its efficacy in suppressing tumor growth and metastasis. The control group displayed numerous irregular pulmonary nodules with rough surface textures, indicating malignant lung metastasis (Figure 7d). Comparative analysis of the metastatic foci confirmed that the TP-SL@siRNA-PB combination therapy was highly effective in treating and suppressing lung metastases (Figure 7e). Notably, the TP-SL@siRNA-PB group exhibited a significant reduction in the number of foci, which is a key indicator of therapeutic efficacy. This group also demonstrated the most effective inhibition of PD-L1, promoting cancer cell apoptosis and triggering immune responses via T cells.

The survival rates of mice bearing B16F10 lung metastases treated with nanoparticles and PD-L1 siRNA were recorded to evaluate the treatment efficacy (Figure 7f). The results demonstrated that TP-SL@siRNA-PB significantly prolonged the survival of B16F10-bearing mice, with a median survival time of 36 days when combined with anti-PD-1 therapy during long-term observation. This extended survival suggests a synergistic effect between TP-SL@siRNA-PB and anti-PD-1 treatment, likely due to enhanced immune modulation and improved tumor suppression. The combination therapy may effectively reduce immune evasion by silencing PD-L1 expression while simultaneously promoting an antitumor immune response.

### 2.12 *In Vivo* GFP-pDNA Delivery by TP-SL@PB

*In vivo* pDNA transfection was assessed to determine the effectiveness of these nanoparticles in delivering pDNA to lung cells. Three days after injecting GFP-pDNA-loaded TP-SL, TP-SL@PB, or TP-SL@IO into the tail vein, the mice were sacrificed, and their tissues sectioned. GFP expression was visualized using CLSM (Figure 7g). The results showed that TP-SL@PB and TP-SL@IO treatments significantly enhanced GFP expression compared to the LNP group, demonstrating superior transfection efficiency that can be attributed to these nanoparticles.

The findings confirm that the nanoparticles, particularly TP-SL@PB and TP-SL@IO, are not only safe but also exhibit enhanced capabilities for intracellular delivery of pDNA to the lungs. The upregulation of GFP expression in these groups highlights their potential for effective genetic cargo delivery, which is a crucial factor in siRNA-based therapeutic strategies. Importantly, the biocompatibility of TP-SL formulations, evidenced by the lack of major organ damage, supports their candidacy for further development in clinical applications. The enhanced transfection efficiency suggests that these nanoparticles can effectively penetrate cellular barriers and deliver therapeutic agents to targeted tissues (Figure 7h). Overall, TP-SL@siRNA-IO stands out as a promising platform, combining safety and efficacy for siRNA delivery. Its ability to achieve robust transfection and therapeutic effects underscores its potential for treating metastatic tumors and advancing the field of RNA-based therapeutics. Furthermore, the organs were harvested and stained with H&E. No significant damage was observed in the mice treated with TP-SL, TP-SL@PB, or TP-SL@IO compared to the control group injected with PBS (Figure S14). This indicates that these nanoparticle formulations are biocompatible and do not cause overt toxicity to vital organs.

### 2.13 T-Cell Infiltration in Lung Metastasis After Treatment with TP-SL@siRNA-PB

For immunotherapy, direct physical contact between T cells and cancer cells is essential for an effective antitumor response. Therefore, T-cell infiltration in lung metastases following treatment with TP-SL@siRNA-PB was investigated. To evaluate the ability of PD-L1 siRNA to elicit an immune response, T-cell infiltration was assessed using immunohistochemical (IHC) staining. Confocal laser scanning microscopy (CLSM) images revealed significant CD8+ T-cell infiltration in tumor regions after treatment with TP-SL@siRNA-PB (Figure 8a). T cells were clearly observed within the tumor microenvironment, indicating successful immune activation. Moreover, TP-SL@siRNA-PB induced a large number of T cells colocalized with cancer cells, demonstrating its effectiveness in targeting lung metastases.

**Figure 8:**
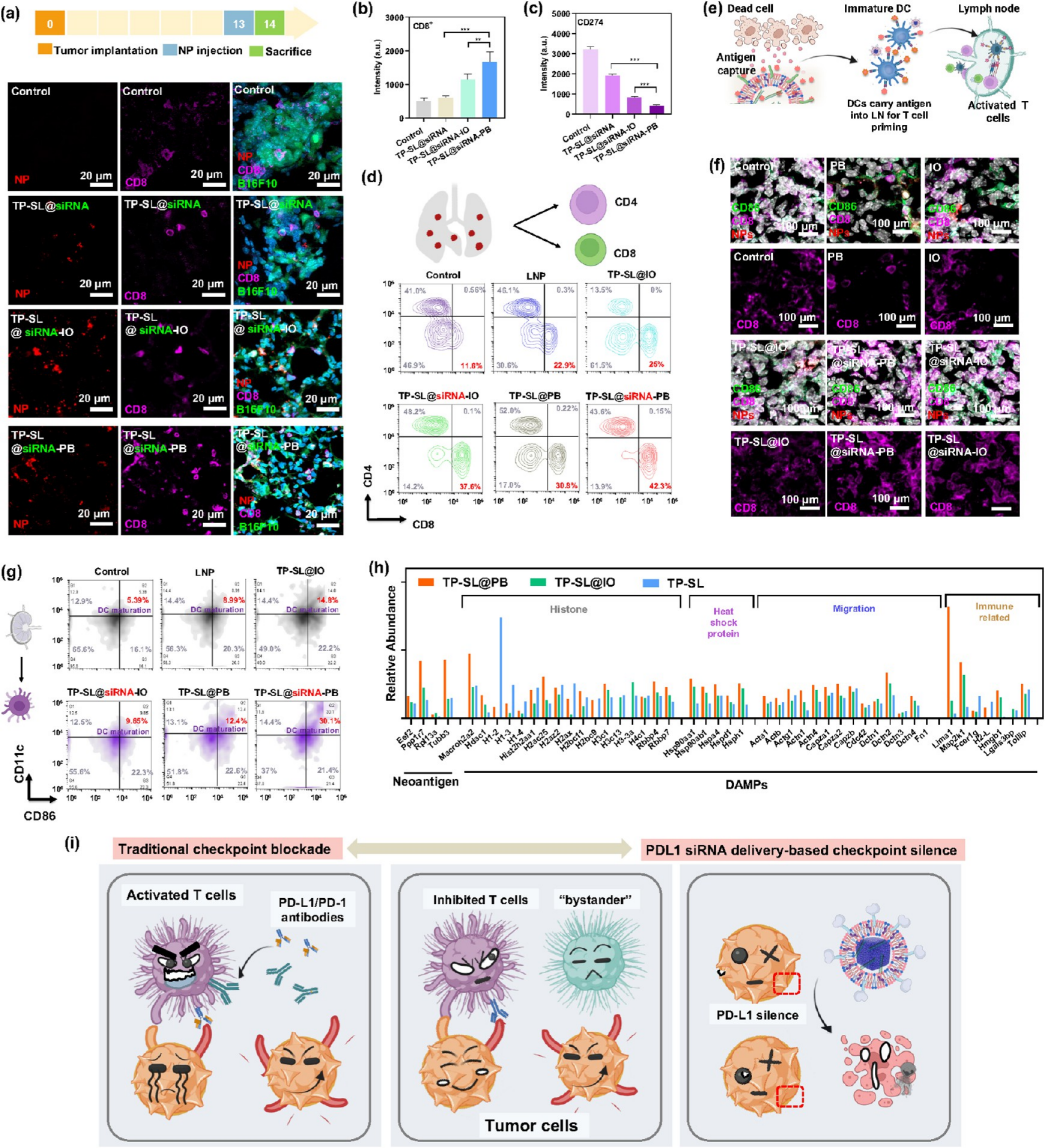
(a) CLSM images of lung metastases treated with TP-SL@siRNA, TP-SL@siRNA-IO, and TP-SL@siRNA-PB at 24 h postinjection, respectively. The quantification of fluorescence images of (b) CD8^+^ T cells and (c) expression of CD274 after various treatments. Error bars represent mean ± SEM, *n* = 3; **p < 0.01, and ***p < 0.001 compared with the group by one-way ANOVA with Tukey’s multiple comparison test. (d) *In vivo* immune response study in lung models by using flow cytometry analysis. (e) Illustration of antigen capture and stimulation for immunotherapy in the lymph node (LN). (f) CLSM images of lymph nodes. White, green, and purple fluorescence represent nucleus staining with DAPI, DCs with CD86, and T cells with CD8. (g) *In vivo* flow cytometry analysis of dissected LN tissue (CD11c and CD86) 24 h post-treatment. (h) Percentage of antigen capture and typical chemokine secretion in B16F10 cell line after TP-SL@PB treatment, quantified by LC-MS/MS. (i) Schematic illustration comparing the conventional checkpoint blockade strategy with the RNAi-driven tumor-targeted checkpoint nanoblocker.

Figure 8b,c presents the quantification results of CD8 and CD274 expression within the tumor areas. The fluorescence signals of CD8 following TP-SL@siRNA-PB treatments were approximately 3-fold higher than those observed in the control groups. This significant enhancement indicates the superior capability of the TP-SL@siRNA-PB system in eliciting a robust T-cell response against the tumor. Such an increase in CD8 levels underscores the system’s potential to modulate the tumor microenvironment and activate cytotoxic T lymphocytes effectively. CLSM images and flow cytometry of CD274 expression levels at the tumor site following various treatments are presented in Figure S15. This regulation is critical as PD-L1 overexpression is a known mechanism by which tumors evade immune surveillance. By silencing PD-L1 expression, TP-SL@siRNA-PB not only disrupts immune evasion but also enhances the effectiveness of subsequent immune therapies including immune checkpoint inhibitors. These findings highlight the dual benefits of the treatment: activating antitumor T-cell responses and modulating immunosuppressive signals.

Flow cytometric analysis was conducted to further evaluate the immune responses in the lungs. Following lung harvest, immune cell populations were quantified. In Figure 8d, both nanoparticle formulations (with and without PD-L1 siRNA) enhanced the expression levels of cytotoxic T cells (CD8+) and helper T cells (CD4+), demonstrating that TP-SL@siRNA-PB effectively activates T cells (The preparation process and the gating strategy of flow cytometry for *in vivo* flow cytometry shown in Figures S16 and S17). The increase in CD8+ T-cell expression was notable, albeit by only a few percentage points. This is also attributed to the efficacy of TP-SL@siRNA-PB treatment in reducing tumor foci and shrinking tumors. As the tumor burden decreases, the recruitment of T cells to the tumor site might also decline, potentially contributing to the observed limited increase in CD8+ levels. Furthermore, the levels of immune factors, including tumor necrosis factor, interferon-γ (IFN-γ), and interleukin-10 (IL-10), in lung tissues treated with various samples were quantified by using ELISA kits. In Figure S18, IFN-γ and IL-12 levels were significantly elevated in the TP-SL@PB and TP-SL@IO groups compared to the control group, indicating a robust immune response.

### 2.14 Dendritic Cell Maturation and Antigen Delivery to Lymph Nodes by TP-SL@PB

The lymphatic system plays a central role in coordinating immune responses across the body. By facilitating the transport of immune cells and signaling molecules, it indirectly shapes immune activity within the lungs.^50,51^ This is particularly relevant in scenarios where robust immune responses in the lungs are critical such as in lung metastasis immunotherapy (Figure 8e). Lymphatic vessels serve as conduits, enabling immune cells to travel from peripheral tissues to lymph nodes and eventually re-enter the bloodstream. This dynamic trafficking is essential for initiating and sustaining effective immune responses.^52,53^ In the context of lung metastasis, a thorough understanding of immune cell migration and its regulation through the lymphatic system is vital.

The effect of TP-SL@PB on *in vivo* recruitment of dendritic cells (DCs) was evaluated in mice bearing B16F10 lung metastases. 24 h postinjection, lymph nodes were harvested, and the populations of DCs and T cells were quantified (Figure 8f). CLSM images of lymph node tissues stained with CD86 (a marker indicative of DC maturation) and CD8+ (a marker of T-cell activation and immune activity) were analyzed. Fluorescence signals were color-coded, with blue representing nuclei stained with DAPI, green indicating CD86+ DCs, and purple marking CD8+ T cells. The results identified four distinct groups within the lymph nodes with significantly higher CD86 expression observed in the TP-SL@PB group. This finding highlights the accumulation of nanoparticles within the lymph nodes, promoting enhanced DC maturation. Notably, the TP-SL@PB group exhibited a 2-fold increase in CD8+ expression compared to the other groups, underscoring its ability to potentiate T-cell activation and enhance the overall immunotherapeutic response. Further flow cytometry analysis corroborated these observations, showing a pronounced increase in DC maturation following treatment with TP-SL@PB (Figure 8g). This dual enhancement of DC and T-cell activity likely contributes to the superior efficacy of the TP-SL@PB formulation in stimulating robust immune responses in lung metastasis models.

Liver and renal functions were evaluated after 24, 48, and 72 h of TP-SL@PB and TP-SL@IO treatments (Figure S19). Key liver function markers, alanine aminotransferase (ALT) and alkaline phosphatase (ALP), were measured. ALT, primarily found in the liver, plays a role in amino acid metabolism, and its elevated levels typically indicate liver damage or disease. ALP, present in the liver, bones, kidneys, and bile ducts, is involved in bile production and bone mineralization. The results indicate that the therapeutic interventions had minimal impact on these organs, demonstrating the safety and compatibility of the antineoplastic treatments with normal organ function.

### 2.15 Antigens Capture and Analysis by TP-SL@PB

The effects of TP-SL, TP-SL@IO, and TP-SL@PB on T-cell recruitment through antigen capture were systematically evaluated. The release of neoantigens and damage-associated molecular patterns (DAMPs) by B16F10 cells was also assessed. Antigen release and subsequent capture by TP-SL, TP-SL@IO, and TP-SL@PB were analyzed using liquid chromatography–mass spectrometry (LC-MS/MS) on an Orbitrap Elite hybrid ion trap-Orbitrap mass spectrometer (Thermo Fisher, Figure S20).^5,54^ In the context of *in vitro* antigen capture, Figure 8h highlights the number of distinct proteins detected on the particles, illustrating the minimal influence of surface charge on antigen binding. Tumor-derived protein antigens (TDPAs) are important components of the human immune surveillance system, especially in cancer immunotherapy. These antigens include tumor-associated antigens (TAAs) and damage-associated molecular patterns, which are released because of tumor cell death. Once captured by antigen-presenting cells (APCs), such as dendritic cells (DCs), these proteins trigger a cascade of immune responses designed to attack cancer cells.^55^

In Figure 8h, comparative analysis among TP-SL, TP-SL@IO, and TP-SL@PB treatments highlighted differential protein expression patterns, including neoantigens, DAMPs, histones, heat shock proteins (HSPs), and proteins involved in cell migration and immune regulation. The analysis showed that the presence of neoantigens indicated enhanced immune recognition after treatment (Figure S21). The generation of neoantigens is crucial for immunotherapeutic strategies because it stimulates T-cell activation.^56^ In this study, the increased relative abundance of neoantigens in TP-SL@PB compared with the control group and TP-SL@IO implies that TP-SL@PB may promote antigen presentation and enhance T-cell-mediated immunogenic response to promote a stronger immunogenic response. The findings are consistent with previous studies showing that iron oxide nanoparticles can modulate immune responses by enhancing the uptake of antigens by dendritic cells.^57,58^ Furthermore, DAMPs, which include proteins such as histones and heat shock proteins (HSPs), play a crucial role in initiating immune responses by signaling tissue damage.^59^ LC-MS/MS results showed that DAMPs were significantly upregulated, especially in the TP-SL@PB treatment group. Histones and HSPs are often released during cellular stress or apoptosis and can promote inflammation by interacting with pattern recognition receptors, such as Toll-like receptors (TLRs) (Table S1).

The elevated levels of DAMPs in the TP-SL@PB suggest that this treatment may induce a form of immunogenic cell death, which could contribute to a more robust antitumor immune response. Histones, particularly H2A and H3 variants, were prominently featured in the LC-MS/MS results, suggesting that chromatin remodeling and epigenetic regulation are impacted by the different treatments. Histones not only serve structural roles in chromatin but are also released into the extracellular space as DAMPs during cell death. The significant elevation of histone levels in the TP-SL@PB group suggests increased cell turnover or damage in tumor cells, leading to the release of nuclear contents that may contribute to the immunogenicity of this treatment. HSPs can also act as DAMPs and have been shown to enhance antigen presentation by chaperoneing peptides to MHC molecules. The enhanced expression of HSPs supports the hypothesis that TP-SL@PB treatment induces cellular stress, contributing to tumor cell death and subsequent immune activation.

Proteins associated with cellular migration, such as actins and cytoskeletal regulators (e.g., actn1, actg1), were differentially expressed across the treatment groups.^60,61^ The TP-SL@PB group exhibited alterations in actin-related proteins, which could affect the cell motility and metastasis potential. Furthermore, immune-related proteins like toll-like interacting protein (Tollip) were observed at higher levels in the TP-SL@PB. Tollip is involved in modulating TLR signaling pathways, and its upregulation suggests enhanced innate immune response activation.^13^ These findings suggest that TP-SL@PB has superior therapeutic potential for treating lung metastases through a combination of direct tumor cytotoxicity and immune activation. Further investigation into the mechanistic pathways activated by TP-SL@PB could elucidate additional therapeutic targets for enhancing antimetastatic therapies.

### 2.16 Immunotherapy with PDL1 Silencing and TP-SL@PB

Immune checkpoint blockers have become a powerful tool for enhancing the host immune response against tumor cells, particularly in advanced cancers. Checkpoint blockade therapy, especially targeting the PD-1/PD-L1 axis, has led to significant clinical progress, with durable responses and prolonged survival in various cancers. This approach typically uses antibodies to block checkpoints on T cells or tumor cells, aiming to restore T-cell-mediated tumor killing. However, sustained clinical benefits have been observed in only a small subset of patients, with challenges such as physiological barriers and limitations of conventional therapies hindering further progress. The therapeutic efficacy is largely dependent on the presence of tumor-killing T cells in tumor tissues.

In our study, a pH-responsive lung metastatic-targeted catalyst containing the tumor penetration polymer (TP)/solid lipids (SL)-coated Prussian blue (TP-SL@PB)-enhanced PD-L1 siRNA delivery and self-cascade antigen capture is developed for reprogramming immunodeficiency. Intravenously injected TP-SL@PB accumulated in the blood vessel-poor lung metastases via the organ-selective targeting and charge conversion of TP. In tumor clusters, SL@PB exerts catalytic and lysosomal escape effects, easily enhancing siRNA delivery and thus downregulating PDL1. Catalysis also promotes the release of tumor-associated antigens (TAAs).

Based on this concept, PD-L1 siRNA delivery can specifically silence both membranous and cytoplasmic PD-L1, rather than merely bind to PD-L1 on the tumor cell membrane (Figure 8i). This approach effectively shuts down the checkpoint-induced immunosuppression and/or drug resistant RNAi-driven tumor-targeted checkpoint nanoblockers can directly induce programmed cell death in H460 cells via the STAT3/caspase apoptosis pathway, without requiring T-cell intervention. This T-cell-independent checkpoint therapy represents a unique and superior approach compared to traditional antibody-based checkpoint inhibitors and current PD-L1 knockdown agents.

## 3 Conclusions

In summary, we synthesized activating lipid-encapsulated inorganic magnetic nanoparticles and demonstrated the success of high-performance siRNA delivery and enhancing the therapeutic effects of PD-L1 siRNA in lung metastatic immunotherapy. The TP-SL@IO nanoparticles exhibited superior efficiency in silencing PD-L1, activating T cells, and boosting immune responses in both cellular and animal models. Intravenous injection of these nanoparticles into mice effectively increased their accumulation in the lungs and suppressed PD-L1 expression in lung metastatic tumor cells, thereby achieving immunotherapeutic effects. The incorporation of magnetic hyperthermia further amplified these effects, in which TP-SL@IO exhibited superior mRNA delivery efficiency, and a greater inhibition of PD-L1 expression in approximately 60% of B16F10 cells compared to LNP. By improving the delivery and stability of PD-L1 siRNA, these nanoparticles significantly boosted immune responses and suppressed tumor growth in both *in vitro* and *in vivo* models. The dual function of generating reactive oxygen species through the Fenton reaction and efficient siRNA delivery offers a promising approach for overcoming resistance to current immunotherapies. These findings underscore the potential of this delivery system for the further exploration of multifunctional nanoplatforms as targeted cancer therapies, particularly for lung cancer patients with metastasis.

## 4 Experimental Section

### 4.1 Synthesis of Prussian Blue Nanocubes (PB)

PB was prepared by using a modified procedure. Briefly, 3 g of PVP (poly­(vinylpyrrolidone), K30) and 226.7 mg of K_3_[Fe­(CN)_6_] (potassium hexacyanoferrate III) were dissolved in 40 mL of deionized water under vigorous magnetic stirring until a clear yellow solution was obtained. Subsequently, 35 μL of concentrated hydrochloric acid (HCl, 12 M) was added, and the mixture was stirred at room temperature for 30 min. The resulting solution was then baked in an oven at 85 °C for 20 h, forming a dark blue solution containing PB nanocubes.

The solution was centrifuged at 12,000 rpm for 10 min, and the pellet was washed three times with 75% ethanol. The collected precipitate was resuspended in 3–5 mL of 75% ethanol and dried at 60 °C overnight to obtain PB single crystals. Finally, the PB adhering to the wall of the sample bottle was scraped off to obtain the final powder product.

### 4.2 Synthesis of Porous IO Nanoparticles

Magnetic porous IO nanoparticles were synthesized from PB via a simple calcination process in a tube furnace. The transformation of PB to IO is divided into two stages. In the first stage, the PB powder was heated from room temperature to 350 °C in air at a heating rate of 2 °C/min for 2 h. At this stage, due to the large temperature gradient, PB partially transformed into magnetic porous IO, mainly in the near-surface region. In the second stage, heating was continued for 3 h and 17 min, with a total duration of 6 h. During this stage, the relatively smooth outer shell of the IO nanoparticles is further transformed into a porous structure.

### 4.3 Synthesis of Selective Organ Targeting (SORT) Lipid Nanoparticles

Stock solutions of 1,2-dioleoyl-*sn*-glycero-3-phosphocholine (DOPC), 1,2-dioleoyl-3-trimethylammonium-propane (DOTAP), 1,2-dimyristoyl-*sn*-glycero-3-phospho-(1′-rac-glycerol) (sodium salt) (DMPG), and methoxy poly­(ethylene glycol) conjugated to a C18 lipid chain (mPEG-C18) were prepared in advance at a concentration of 10 mg/mL by dissolving them in ethanol. A variety of phospholipids with distinct charges were screened, including neutral DOPC, anionic DMPG, cationic DOTAP, and mPEG-C18, using a lipid-to-mPEG-C18-to-cholesterol ratio of 5:1:4. For the preparation of positively charged lipid nanoparticles (LNPs) targeting the lung (Lung SORT), 0.873 mg of DOTAP, 1.284 mg of mPEG-C18, and 0.387 mg of cholesterol (molar ratio: 5:1:4) were dissolved in ethanol and mixed thoroughly. The solution was evaporated at room temperature to form a thin lipid film. The lipid film was then hydrated with 500 μL of phosphate-buffered saline (PBS, pH 7.4) and subjected to sonication to obtain LNPs at a final concentration of 5 mM. To prepare solid lipid nanoparticles (SLNs), the hydrated lipid mixture was rapidly cooled in an ice bath while being sonicated, promoting the solidification of lipids into nanoparticles.

For neutral LNPs targeting the liver (Liver SORT), 0.393 mg of DOPC, 1.284 mg of mPEG-C18, and 0.677 mg of cholesterol (molar ratio: 2:1:7) were dissolved in ethanol and mixed. After solvent evaporation, the lipid film was hydrated with 500 μL of PBS (pH 7.4) and sonicated to obtain LNPs. The SLNs were formed by cooling the hydrated lipid suspension in an ice bath during sonication, ensuring the solidification of the lipid matrix. For the preparation of negatively charged LNPs targeting the spleen (Spleen SORT), 0.517 mg of DMPG, 1.284 mg of mPEG-C18, and 0.580 mg of cholesterol (molar ratio: 3:1:6) were dissolved in ethanol and mixed. Following solvent evaporation, the lipid film was hydrated with 500 μL of PBS (pH 7.4) and sonicated to obtain LNPs. The SLNs were then prepared by immediately cooling the hydrated lipid suspension in an ice bath while maintaining sonication, allowing the lipid components to solidify into stable nanoparticles. All prepared SLN formulations were stored at 4 °C until further use.

### 4.4 Synthesis of Lipid-Coated Nanoparticles with Plasmid DNA or PD-L1 siRNA

The lung-targeting TP-SL@PB and TP-SL@IO were prepared using a method similar to lipid nanoparticle formulation. Stock solutions of Prussian Blue (PB) and iron oxide (IO) nanocubes were prepared in advance at a concentration of 2 mg/mL in 95% ethanol. To prepare TP-SL@PB, 0.873 mg of 1,2-dioleoyl-3-trimethylammonium-propane (DOTAP), 1.284 mg of methoxy poly­(ethylene glycol)-aldehyde (mPEG-CHO), and 0.387 mg of cholesterol (molar ratio: 5:1:4) were dissolved in ethanol. Then, 0.172 mg of PB nanocubes was added to the lipid mixture and thoroughly mixed. The solution was evaporated at room temperature to form a thin lipid film. The film was then hydrated with 500 μL of phosphate-buffered saline (PBS, pH 7.4) and subjected to sonication to obtain TP-SL@PB. A similar procedure was followed for TP-SL@IO, substituting PB nanocubes with IO nanocubes. After sonication, the prepared TP-SL@PB or TP-SL@IO was centrifuged at 12,000 rpm for 10 min to remove lipid nanoparticles that did not contain PB or IO.

The PD-L1 siRNA was purchased from Dharmacon, with target sequences from the siGENOME Mouse Cd274 siRNA SMARTpool as follows: GAAUCACGCUGAAAGUCAA, GAGCCUCGCUGCCAAAGGA, GAGGUAAUCUGGACAAACA, and GAUAUUUGCUGG CAUUAUA. Before use, the unopened siRNA (10 nmol) was briefly centrifuged to collect the contents at the bottom of the tube and then resuspended in 500 μL of RNase-free distilled water to prepare a 20 μM stock solution. The TP-SL@PB or TP-SL@IO nanoparticles were then combined with siRNA through electrostatic adsorption, leveraging the interaction between negatively charged ribonucleic acid and positively charged lipid nanoparticles.

### 4.5 Characterizations

The morphologies of Prussian Blue (PB) nanocubes, TP-SL@PB, iron oxide (IO) nanoparticles, and TP-SL@IO were examined by using field emission scanning electron microscopy (FE-SEM, JSM-7000F, JEOL). For SEM analysis, all samples were suspended in ethanol, dropped onto silica wafers, and dried overnight in an oven. The dried samples were then coated with a thin platinum film to enhance the conductivity for improved detection during electronic sputtering. The morphologies and elemental mapping of PB, TP-SL@PB, IO, and TP-SL@IO were further analyzed using Field Emission Transmission Electron Microscopy (FE-TEM, JEM-F200, JEOL). For TEM analysis, all samples were dispersed in deionized (DI) water, dropped onto a 200 mesh copper grid, and air-dried. Lipid-coated nanoparticles underwent negative staining with 2 wt % phosphotungstic acid hydrate for 4 min to enhance lipid visualization. After the sample was dried, TEM imaging was performed, and elemental mapping was analyzed using the energy-dispersive spectroscopy (EDS) system.

The size distribution and ζ-potential of the nanoparticles were measured by using dynamic light scattering (DLS, Zetasizer Ultra Red training, Malvern). For DLS analysis, all samples were dispersed in DI water and diluted to a lightly translucent solution in a plastic cuvette. The size distribution was determined based on Brownian motion, while the ζ-potential was measured via electrophoresis. The binding energy of the nanoparticles’ surfaces was assessed using high-resolution X-ray Photoelectron Spectroscopy (HRXPS, PHI Quantera II, ULVAC-PHI). PB nanocubes and IO nanoparticles were dispersed in ethanol and repeatedly deposited onto 7 mm × 7 mm silica wafers until a uniform coating was achieved. The crystal structure of PB nanocubes and IO nanoparticles was characterized by using powder X-ray diffraction (XRD, APEX DUO, Bruker). The thermal stability of PB, TP-SL@PB, IO, and TP-SL@IO was analyzed using Thermogravimetric Analysis (TGA, 2-HT, Mettler-Toledo). Lipid-coated nanoparticles were freeze-dried to obtain powdered samples before thermal analysis.

### 4.6 *In Vitro* ROS Generation by Particles

O-phenylenediamine (OPD), which is a kind of ROS detector, was used to detect the catalytic ability of the ROS generation from PB and IO nanoparticles. ROS (reactive oxygen species), including peroxide ion (O_2_^2–^), hydroxyl radical (^•^OH), and so on, can cause irreversible damage to DNA of cancer cells. Colorless OPD is oxidized to yellow dimer by ^•^OH of Fe_2_^+^ and Fe_3_^+^ in PB and IO nanoparticles via the Fenton reaction. The 5 mM OPD solution, 25 mM H_2_O_2_, and 1 mg/mL nanoparticles were prepared in PBS solution in advance. The final solution was prepared as 0.5 mM OPD and 100 μg/mL nanoparticles containing 2.5 mM H_2_O_2_, and the nanoparticles were added last. After 20 min, the colorless solution transformed into obviously yellow. Finally, the solution containing oxidant OPD showed the characteristic adsorption peak by ultraviolet–visible (UV–vis) Spectrometer (SP-8001, Metertech).

For *in vitro* study of ROS generation, the commercialized detection assay kit was utilized to confirm the production of ROS. Before adding the nanoparticles, the B16F10 cells were cultured in confocal dishes with a concentration of 2 × 10^5^ cells/mL in 1 mL medium overnight to allow the cells become adherent. According to the guideline, 250× OH580 stain stock solution should be prepared by adding 50 μL of DMSO in the vial of OH580, then 25 μL of 250× OH580 stain stock solution was diluted with assay buffer to 10 mL to prepare the stain working solution. The culture medium had to be removed before adding 1 mL of the stain working solution. After placing the cells in the incubator for 1 h, 100 mg/mL of the nanoparticles were added for another 1 h. Finally, the cells were washed with warm PBS three times to remove the remaining nanoparticles and stained with 2 μg/mL Hoechst 33342 dying buffer for 30 min. After washing with warm PBS three times and replacing the solution with the assay buffer, the samples could be observed for the production of ROS by CLSM at 540/590 nm excitation/emission.

### 4.7 Cellular Culture and Cytotoxicity

The B16F10 cell line is derived from the skin tissue of C57BL/6J mice with melanoma, while the NIH/3T3 cell line is a fibroblast cell line derived from the NIH/Swiss mouse embryo. Both cell lines were maintained in 10 cm cell culture dishes containing 10 mL of Dulbecco’s modified Eagle’s medium (DMEM), supplemented with 10% fetal bovine serum (FBS) and 1% Penicillin-Streptomycin-Amphotericin B (PSA antibiotics). Cells were cultured at 37 °C in a 5% CO_2_ incubator. The PSA was used to prevent microbial contamination. The culture medium was refreshed every 2–3 days or when the cells reached near-confluence.

For subculturing, the original medium was removed, and the cells were washed twice with 5 mL of prewarmed PBS. After removing any residual PBS, 1 mL of 0.25% trypsin-EDTA was added to detach the cells from the culture dish. The dish was incubated at 37 °C for 3 min to allow the trypsin to break down the proteins holding the cells to the surface. Once the cells had dispersed, 2 mL of fresh medium was added to neutralize the trypsin. The suspension was then centrifuged at 800 rpm for 5 min. After the cells were resuspended in 3 mL of medium, 200 μL of the cell suspension was seeded into a new dish containing 10 mL of fresh medium. For cell counting, 20 μL of the cell suspension was mixed with 80 μL of trypan blue and loaded into the chambers of a hemocytometer. Cell concentration was determined by using a microscope.

For the maintenance of the GFP-P2A-NanoLuc B16F10 cell line, which is a B16F10 cell line modified with a green fluorescence protein (GFP) gene and a luciferase gene, the procedures were the same as those for the wild-type B16F10 cells. The only difference was that the DMEM used for maintenance contained 0.1% puromycin, an antibiotic used to select cells that express the GFP and luciferase genes.

To evaluate the cytotoxicity of the nanoparticles, PrestoBlue assay was performed. On day 1, cells were seeded into a 96-well plate at a density of 1 × 10^5^ cells/mL, with 100 μL of cell suspension per well. PBS was added to the outer wells of the plate to prevent medium evaporation. On day 2, nanoparticles were added to the wells at various concentrations. The nanoparticles were suspended in medium and serially diluted to concentrations of 200, 100, 50, 25, and 0 μg/mL, with each concentration tested in five wells. After 24 h, 20 μL of PrestoBlue solution was added to each well, and the plate was incubated for 10 min at 37 °C. The fluorescence intensity was measured at an emission wavelength of 530 nm using a microplate reader to assess cell viability. The fluorescence intensity of the control group (0 μg/mL) was considered 100% cell viability, and the fluorescence intensity of the nanoparticle-treated groups was compared to that of this control to calculate cell viability.

### 4.8 Cellular Uptake Analysis

To assess the potential of nanoparticles entering cells, the following procedure was carried out. On day 1, 12 mm coverslips were coated with positively charged poly-l-lysine (PLL) to enhance cell adhesion. The coverslips were cleaned with alcohol to remove any oil stains and then exposed to UV light in a laminar flow hood for 30 min to sterilize them. Each coverslip was then coated with 100 μL of PLL for 15 min. Afterward, the coverslips were washed with sterile PBS and placed in a 24-well plate. Next, B16F10 cells were cultured on the coverslips at a concentration of 1 × 10^5^ cells/mL in 500 μL of medium per well. Prior to use, the lipid nanoparticles were stained with DiI dye, and the ethanol was evaporated during the preparation of the nanoparticles. On day 2, the DiI-stained lipid nanoparticles were dispersed in the medium and incubated with the cells. Two experimental groups were set up to evaluate cellular uptake: a concentration-dependent group and a time-dependent group. The concentration-dependent group aimed to determine how the nanoparticle concentration affected cellular uptake, while the time-dependent group was designed to investigate the kinetics of nanoparticle endocytosis.

After incubation, the cells were fixed with 3.7% formaldehyde for 15 min and then stained with F-actin and DAPI for 2 h 30 min, respectively. PBS was used to rinse the cells between each staining step. Finally, the cells on the coverslips were mounted using a mounting medium (without DAPI) and sealed with nail polish. The samples were observed using CLSM (Carl Zeiss, LSM800, Oberkochen, Germany).

For flow cytometry (Attune NxT Flow Cytometer, Thermo Fisher Scientific) analysis, the coverslips were not required. Instead, B16F10 cells at a concentration of 5 × 10^4^ cells/mL were cultured in 2 mL of medium per well in a 6-well plate. The procedure was similar to the CLSM protocol, with DiI-stained lipid nanoparticles dispersed in the medium and incubated with the cells. After the incubation, the cells were collected for flow cytometry analysis. To collect the cells, 0.5 mL of trypsin was added per well, and the plate was incubated for 3 min to detach the cells. One milliliter of medium was added to neutralize the trypsin reaction. The cells were then collected into 15 mL centrifuge tubes and centrifuged at 800 rpm for 5 min. After removing the trypsin, the cells were resuspended in 1 mL PBS and transferred to flow cytometry tubes for measurement.

### 4.9 Plasmid DNA (pDNA) Transfection

To evaluate the transfection efficiency of the nanoparticles, plasmid DNA with GFP was loaded in lipid nanoparticles. The lipid nanoparticles and pDNA were prepared with different ratios by mixing via electrostatic adsorption between negatively charged ribonucleic acid and positively charged lipid nanoparticles, and they were dispersed in serum-free medium to prevent pDNA from adsorbing with protein. Instead, the negative charge pDNA is absorbed onto negatively charged PB and IO based on the cation bridge construction and the electric double layer formation by weaker electrostatic repulsion.^62^ B16F10 cells were seeded in a 24-well cell culture plate at a density of 2.5 × 10^4^ cells per well overnight. After attaching, the cells were incubated with 500 uL of pDNA-loaded lipid nanoparticles in serum-free medium. After 6 h, 500 uL 10% FBS medium was added to the wells to maintain cell growth for 48 h. Finally, the cells were fixed with 3.7% formaldehyde for 15 min and stained with DAPI for 30 min. The transfection efficiency of the nanoparticles was examined for GFP expression by CLSM.

### 4.10 PD-L1 siRNA-Mediated Protein Knockdown

To evaluate the feasibility of nanoparticles coated with lipid nanoparticles to knockdown PD-L1 expression in B16F10 cells, immunofluorescence staining of PD-L1 expression in B16–F10 cells was utilized. On the first day, B16F10 cells were seeded on coverslips in a 24-well cell culture plate at a density of 2.5 × 10^4^ cells per well overnight. On the second day, before adding nanoparticles with siRNA, 10 μg of nanoparticles and 1 μg of siRNA were mixed in advance and dispersed in serum-free medium. The original medium was removed and the cells were incubated with siRNA-loaded lipid nanoparticles in serum-free medium. After 6 h, 500 uL 10% FBS medium was added in the well to maintain cell growth for 48 h. On the fourth day, the cells were fixed with 3.7% formaldehyde for 15 min. Then, 0.1% triton was added to lyse the cells for the following combination with antibodies. After 15 min, triton was removed and washed with PBS three times. After that, 5% bovine serum albumin (BSA) was added. Waiting for 1 h, 0.5 mL BSA containing 0.1% PD-L1 antibodies was added for 2 h. After removing PD-L1 antibodies and washing with PBS twice, 0.1% goat antirat Alexa Fluor 647 secondary antibody in BSA was added for staining 1 h. Finally, the cells on the coverslips were sealed with DAPI mounting and nail polish. The PD-L1 expression of B16F10 cells was observed by CLSM.

## Supplementary Material



## Notes

Functions of each protein (Table S1); various formulation of solid lipid nanoparticles (Figure S1); *in vivo* IVIS images of key clearance organs from lung metastasis tumor-bearing mice after treating by various formulations (Figures S2–S4); stability study of hydrated dynamic particle size and polydispersity index (PDI) variation over the time of TP-SL@PB in distilled water or PBS (Figure S5); UV–vis adsorption spectra of the catalyzed oxidation of o-phenylenediamine of PB with various concentration of H_2_O_2_ (Figure S6); CLSM images of *in vitro* assessments of HMGB1 after treatment with TP-SL@PB and TP-SL@IO (Figure S7); CLSM images of lysosomes of B16F10 cells after treatment with TP-SL@PB (Figure S8); CLSM images of lysosomes in B16F10 cells after incubation with TP-SL@PB (Figure S9); CLSM images of DCs incubated with TP-SL, TP-SL@PB, and TP-SL@IO (Figure S10); SEM images of B16F10 cells treated by TP-SL and TP-SL@PB (Figure S11); TEM and energy-dispersive X-ray spectroscopy (EDS) mapping images of TP-SL@siRNA-PB (Figure S12); western blot of PD-L1 expression of B16F10 cells (Figure S13); hematoxylin and eosin (H&E) staining of tissues from glioma-bearing mice (Figure S14); CLSM images and flow cytometry of CD274 expression of lung metastases treated with TP-SL@siRNA-PB (Figure S15); the preparation process for *in vivo* flow cytometry (Figure S16); gating strategy of flow cytometry (Figure S17); concentrations of immune factors (Figure S18); biochemical indices of liver and kidney (Figure S19); experimental procedures for analyzing neoantigens and damage-associated molecular patterns (DAMPs) from B16F10 cells (Figure S20); and release of neoantigens and damage-associated molecular patterns (DAMPs) from B16F10 cells following particle treatment was analyzed (Figure S21) (PDF)
