# Supplementary material for: Reprogramming Immunodeficiency in Lung Metastases via PD-L1 siRNA Delivery and Antigen Capture of Nanosponge-Mediated Dendritic Cell Modulation
Source: ACS Nano. 2025 Jul 5;19(27):25134–53. doi: 10.1021/acsnano.5c05395 (PMC12269356; doi:10.1021/acsnano.5c05395)
Supplement: Supplementary file 1 [file nn5c05395_si_001.pdf]

Supporting Information

Reprogramming Immunodeficiency in Lung Metastases *via* PD-L1 siRNA Delivery and Antigen Capture of Nanosponges-Mediated Dendritic Cell Modulation

Thi My Hue Huynh[1], Pin-Xuan Huang[1], Kang-Li Wang[1], Ngoc-Tri Tran[1], Hoi Man Iao[1], Wan-Chi Pan[1], Yun-Hsuan Chang[1], Hui-Wen Lien[1,2], Alan Yueh-Luen Lee[2], Tsu-Chin Chou[3], Wen-Hsuan Chiang[4], Shang-Hsiu Hu[1,3,*]

[1]Department of Biomedical Engineering and Environmental Sciences, National Tsing Hua University, Hsinchu 300044, Taiwan.

[2]National Institute of Cancer Research, National Health Research Institutes, Miaoli County 35053 Taiwan.

[3] Institute of Analytical and Environmental Sciences, National Tsing Hua University, Hsinchu 300044 Taiwan.

[4]Department of Chemical Engineering, National Chung Hsing University, Taichung 402 Taiwan

Experimental Section

siRNA loading efficiency and release kinetic

To evaluate the siRNA loading efficiency (EE%) and release profile in TP-SL@PB, the amount of siRNA incorporated into the nanoparticles was measured relative to the initial amount used for loading. Encapsulation efficiency (EE) is defined as the percentage of the nanoparticle's total weight attributable to the loaded siRNA. For quantification, siRNA was labeled with Cy3 and measured using fluorescence spectrophotometry (Hitachi U-2900) at the Cy3-specific wavelength (excitation peak at 554 nm and an emission peak at 568 nm), based on a pre-established calibration curve. The release profile of siRNA was similarly assessed using fluorescence spectrophotometry at the corresponding Cy3 wavelength. Encapsulation efficiency was then calculated using the appropriate equation.

$$\text{Encapsulation efficiency (EE\%)} = \frac{siRNA\ (weight) - siRNA\ in\ supernatant(weight)}{siRNA\ (weight)} \times 100\%$$

DC cell uptake and maturation

DC2.4 cells, a murine dendritic cell line, are cultured under standard conditions to maintain viability and functionality. To begin, quickly thaw a frozen vial of DC2.4 cells in a 37 °C water bath. Transfer the cells to a sterile 15 mL conical tube containing 10 mL of prewarmed complete culture medium (RPMI-1640 supplemented with 10% fetal bovine serum (FBS), 1% penicillin-streptomycin, and 50 μM β-mercaptoethanol). Centrifuge at 300g for 5 min, discard the supernatant, and resuspend the pellet in fresh complete medium. Plate the cells in a T-75 flask at a density of 0.5–1 × 10^6 cells/mL and incubate at 37 °C in a humidified 5% CO_2 atmosphere. For routine maintenance, change the medium every 2–3 days and monitor cell confluency. When confluency reaches 70–80%, subculture the cells by gently detaching them with a cell scraper or pipetting, then reseed at a lower density. For experimental setups, seed DC2.4 cells in appropriate culture vessels (e.g., 6-well plates) and allow them to adhere and recover for 24 hours before treatment.

DC2.4 cells are seeded in appropriate culture vessels and treated as needed before staining. For surface marker staining (CD80), cells are washed with PBS, blocked with 1% BSA in PBS for 15 min, and incubated with fluorophore-conjugated anti-CD80 antibody for 30 min at 4 °C in the dark. For intracellular iNOS staining, cells are fixed with 4% paraformaldehyde for 15 min, permeabilized with 0.1% Triton X-100 or a saponin-based buffer, blocked, and incubated with an anti-iNOS primary antibody followed by a fluorophore-conjugated secondary antibody. After washing, cells are resuspended in PBS for flow cytometry analysis or mounted with antifade medium for fluorescence microscopy. Appropriate isotype controls and unstained samples are included for gating and compensation adjustments.

Membrane Disruption Property of TP-SL

To evaluate the effectiveness of DAPI permeation, a modified membrane disruption test was performed according to the procedure described by Kuroda *et al.*[1] First, 105 B16F10 cells per ml were cultured in six-well plates at 37 °C. After 24 h, TP-SL, TP-SL@PB, and TP-SL@IO were added to fresh culture medium and incubated with cells for 30 min. After 2 h of incubation, the medium containing these materials was discarded, and the cells were gently washed 1–2 times with PBS to remove excess particles.

To assess membrane disruption, cells were incubated with DAPI (1 μg mL^{-1}) for 30 min to allow the dye to permeate the damaged membrane. DAPI is a membrane-impermeable dye that selectively stains nuclei with disrupted membranes, indicating compromised membrane integrity. After incubation, cells were gently washed with PBS to remove excess dye. Finally, cell viability and membrane integrity were further assessed using the LIVE/DEAD™ Viability/Cytotoxicity Kit, and fluorescence was observed under a confocal microscope to distinguish live cells from cells with damaged membranes.

Multicellular Spheroids Chip Fabrication

To fabricate a multicellular spheroid chip, a microfluidic hanging drop chip was designed using a straightforward laser-cutting process on a poly(methyl methacrylate) (PMMA, Sigma–Aldrich) sheet. The chip consisted of two layers, precisely cut to create 96 open wells, each with a diameter of 1.0 mm. To improve coating quality and enhance cell–cell adhesion, the bottom layer of the chip was coated with 100 μL of poly(2-hydroxyethyl methacrylate) (pHEMA, BioReagent, powder, suitable for cell culture) dispersed in 95% ethanol at a concentration of 60 mg mL^{-1}.[2] This coating process was performed after treating the coverslip substrate with oxygen plasma. Next, the hydrophilic double-layer chip and the bottom microchannel replicas were carefully aligned and bonded using oxygen plasma treatment. To ensure permanent bonding, the assembled chip was placed in an oven at 65 °C for 24 hours, resulting in the final multicellular spheroid chip.

Western blotting

For Western blotting, load 70 µg of total protein per well onto a 10% SDS-PAGE gel and run under standard electrophoresis conditions using 1× SDS running buffer. After transfer to a PVDF or nitrocellulose membrane, block the membrane in 5% non-fat milk or BSA in TBST for 1 hour at room temperature. Incubate overnight at 4°C with primary antibodies: anti-PD-L1 (GTX638348) and anti-Beta-actin (GTX109639), appropriately diluted in blocking buffer. Wash the membrane in TBST and incubate with the secondary antibody, anti-rabbit IgG (AB_2721865), for 1 hour at room temperature. Following additional washes, detect the protein bands using enhanced chemiluminescence (ECL) and image with a suitable imaging system.

In Vivo Biodistribution Analysis by IVIS, Flow Cytometry, and Tissue Section

The lung metastasis model was established in female C57BL/6J mice (8–10 weeks old) purchased from BioLASCO Taiwan Co., Ltd. The animal use and experimental protocols were approved by the Institutional Animal Care and Use Committee (IACUC) of National Tsing Hua University (Approval No. 110080). To induce lung metastases, GFP-P2A-nanoLUC B16F10 cells (5×10^6 cells/mL in PBS) were injected into the bloodstream via tail vein injection. Each mouse received a 100 µL cell suspension using a 27-gauge needle. To confirm the delivery of lipid nanoparticles to the lungs, DiI-loaded lipid nanoparticles were administered *via* tail vein injection and analyzed using an In Vivo Imaging System (IVIS). Thirteen days post-tumor inoculation, 100 µL of DiI-loaded lipid nanoparticles (2.5 mg/mL) were injected intravenously. After 24 hours, the mice were sacrificed through perfusion with PBS and paraformaldehyde (PFA). Major organs, including the heart, liver, spleen, lungs, and kidneys, were collected, and IVIS was used to measure fluorescence intensity, assessing nanoparticle distribution.

Two methods were employed to distinguish nanoparticle localization between tumor and non-tumor sites: immunofluorescence staining and flow cytometry. For immunofluorescence staining, lung

tissues were fixed in 4% PFA overnight, embedded in OCT tissue freezing medium within 12×12×12 mm disposable molds, and stored at -20 °C. Frozen tissue sections (10 μm thick) were prepared using a cryostat and mounted on polysine-coated microscope slides. The sections were fixed in methanol at -20 °C, washed three times with PBS to remove OCT residue, and encircled with a liquid blocker pen. To prevent non-specific antibody binding, slides were blocked with 5% BSA in PBS for 1 hour. For blood vessel staining, the sections were incubated overnight at 4 °C with a CD31 antibody (BD Pharmingen, #550274, 1:1000 in 5% BSA). After washing off unbound primary antibodies, sections were stained with a goat anti-rat Alexa Fluor 647 secondary antibody (Jackson ImmunoResearch, #112-605-167, 1:1000 in 5% BSA). Finally, tissue sections were mounted with DAPI-containing mounting medium, sealed with nail polish, and analyzed using confocal laser scanning microscopy (CLSM) to assess nanoparticle distribution in tumor and non-tumor areas.

In Vivo Analysis Immune Response Study by Immunofluorescence Staining

The animal model was established using the same method described for biodistribution. For treatment, TP-SL@PB and TP-SL@IO loaded with PD-L1 siRNA were administered on Days 6 and 9, and the mice were sacrificed on Day 14. To assess the immune response in the lungs and immune system, lung and cervical lymph nodes were collected post-sacrifice. Tissue freezing and sectioning followed the previously described protocol. Lung sections were stained with rabbit anti-CD8 primary antibody (Abcam, #217344, 1:1000) and rat anti-CD274 (PD-L1) primary antibody (BioLegend, #124302, 1:500). Lymph node sections were stained with various primary antibodies to analyze different immune cell populations, including rabbit anti-CD8 (Abcam, #217344, 1:1000) and rat anti-CD4 (Abcam, #ab25475, 1:1000) for T cells, as well as rat anti-CD86 (Abcam, #119857, 1:1000) and rabbit anti-CD11c (Abcam, #ab219799, 1:1000) for dendritic cells.

Following primary antibody incubation, lung sections were stained with goat anti-rabbit Alexa Fluor 647 secondary antibody (Jackson ImmunoResearch, #111-605-144, 1:1000) for CD8 and goat anti-rat Alexa Fluor 647 secondary antibody (Jackson ImmunoResearch, #112-605-167, 1:1000) for CD274. Lymph node sections were stained with goat anti-rabbit Alexa Fluor 647 secondary antibody (Jackson ImmunoResearch, #111-605-144, 1:1000) for CD8, goat anti-rat Alexa Fluor 488 secondary antibody (Jackson ImmunoResearch, #112-545-143, 1:1000) for CD4, goat anti-rat Alexa Fluor 647 secondary antibody (Jackson ImmunoResearch, #112-605-167, 1:1000) for CD86, and goat anti-rabbit Alexa Fluor 488 secondary antibody (Abcam, #ab150081, 1:1000) for CD11c. All antibodies were diluted in 5% BSA.

In Vivo Analysis of Immune Response by Flow Cytometry

For flow cytometry analysis, mice were sacrificed via CO_2 inhalation, and their lungs were collected. The lung tissues were cut into approximately 1×1 mm pieces and incubated in 3 mL RBC lysis buffer (eBioscience™ 1x RBC Lysis Buffer, Invitrogen) to lyse erythrocytes. To dissociate the tissues into single cells, a digestion mixture containing 1 mg collagenase (Sigma-Aldrich), 40 μL Dispase I (Sigma-Aldrich), and 20 μL DNase (Sigma-Aldrich) was added. The samples were incubated at room temperature for 60 minutes on an orbital shaker at 190 rpm. After enzymatic digestion, the cell suspension was filtered through a 70 μm cell strainer to remove tissue clumps, transferred to fresh tubes, and centrifuged at 800 g for 8 minutes. The supernatant was discarded, and the cell pellet was resuspended in 5 mL HBSS. To block non-specific binding, 10 μL purified rat anti-mouse CD16/CD32 (Mouse BD Fc Block™) and 50 μL goat serum were added, and the solution was incubated at room temperature for 45 minutes.

Next, 1.5 mL of the blocked cell suspension was transferred to flow cytometry tubes, and 2 μL of CD31 antibody (BD Pharmingen, #550274) was added to each sample, followed by a 1-hour

incubation at room temperature. Subsequently, 2 μL of goat anti-rat Alexa Fluor 647 secondary antibody (Jackson ImmunoResearch, #112-605-167) was added, and the samples were incubated for another hour at room temperature. Finally, all samples were centrifuged at 1,550 rpm for 5 minutes, resuspended in 1 mL PBS, and analyzed by flow cytometry.

The establishment of the animal model followed the same method as described for immunofluorescence staining, with the only difference being the use of B16F10 cells. TP-SL@PB and TP-SL@IO loaded with PD-L1 siRNA were administered on Days 6 and 9, and the mice were sacrificed on Day 14 to examine the immune response via flow cytometry. The pre-processing steps for flow cytometry were the same as those described for biodistribution, differing only in the final staining stage. For lung and lymph node samples, 2 μL of each of the following antibodies were added and incubated at room temperature for 1 hour: PE/Cyanine7 anti-mouse CD45 antibody (BioLegend, clone: 30-F11, #103114) for immune cells, FITC anti-mouse CD3ε antibody (BioLegend, clone: 145-2C11, #100306) for T cells, PE anti-mouse CD4 antibody (BioLegend, clone: GK1.5, #100408) for helper T cells, and APC anti-mouse CD8 antibody (BioLegend, clone: 53-6.7, #100712) for cytotoxic T cells. For dendritic cell staining, FITC anti-mouse CD86 (GL1) (Proteintech, #65068) and APC anti-mouse CD11c (N418) (Proteintech, #65130) were used. Finally, all samples were centrifuged at 1550 rpm for 5 minutes, resuspended in 1 mL PBS, and analyzed by flow cytometry.

Immunofluorescence of Lymph Node Sections

In this study, inguinal lymph nodes (LNs) were harvested from tumor-bearing mice on Day 14, following 24 hours of treatment with DiI-labeled TP-SL, TP-SL@PB, and TP-SL@IO. The collected LNs were fixed in 4% PFA overnight, embedded in OCT, and sectioned into 10 μm thick slices using a cryotome. Next, the LN sections were dehydrated, fixed in 100% methanol for 10 minutes, and washed three times with PBS to remove any residual OCT compound. The sections were then

incubated with 5% BSA blocking and dilution buffer at room temperature for 1 hour before being incubated overnight at 4°C with the appropriate primary and secondary antibodies. These included rat anti-CD86 (1:200 dilution, BD Pharmingen, #550274) followed by anti-rat Alexa Fluor 488 (Jackson ImmunoResearch, #112-545-143) and rabbit anti-CD8 (1:1000 dilution, Abcam, #ab217344) followed by anti-rabbit Alexa Fluor 647 (Abcam, #ab150075). Finally, the immunofluorescence of LN sections was analyzed using confocal laser scanning microscopy (CLSM) after mounting the samples in Fluoroshield mounting medium (Abcam, #ab104139).

In Vivo Anticancer Efficacy and Survival Rate

To evaluate biodistribution and survival rates in the context of synergistic immunotherapy, mice were injected with 5×10^5 B16F10 cells via the tail vein to establish a metastatic lung cancer model. After seven days of tumor growth, the animals were randomly divided into four groups and intravenously injected with 100 μL of a solution containing DiI-labeled particles. The major organs were harvested on Days 7, 15, and 20 post-tumor inoculations. Lung tumor foci were manually counted and further validated using ImageJ software for accuracy. Additionally, survival rates for all groups were recorded daily from the day of B16F10 cell implantation.

Antigen Capture by TP-SL@PB

The zeta potential of the nanoparticles after antigen capture was assessed using dynamic light scattering (DLS) with a Nano-90 Treksizer instrument from Taiwan. To identify the proteins bound to the nanoparticles, samples were analyzed using an Orbitrap Elite™ Hybrid Ion Trap-Orbitrap Mass Spectrometer (Thermo Scientific, USA). Prior to mass spectrometry analysis, the samples were processed using sodium dodecyl sulfate polyacrylamide gel electrophoresis (SDS-PAGE). This involved preparing a 12% separating gel and a 5% stacking gel solution, which were poured into a

glass chamber and leveled with ethanol. After solidification, a comb was inserted to create sample-loading wells, and the gel was stored at 4 °C.

Table S1. The functions of each protein.

Protein	Protein Type	Functions	Neoantigen Potential	DAMP Activity	T Cell Activation & DC Maturation
**Hsp90aa1**	Heat Shock Protein	Chaperone, antigen presentation, immune stimulation	High	Yes	Enhances immune response [3]
**Hsp90ab1**	Heat Shock Protein	Protein folding, immune modulation	High	Yes	Promotes antigen presentation [3]
**Hspd1**	Heat Shock Protein	Mitochondrial stress response, chaperone	High	Yes	Enhances immune activation
**Hsph1**	Heat Shock Protein	Stress response, chaperone	High	Yes	Supports immune cell activation
**Fn1**	Extracellular Matrix	Cell adhesion, migration, immune signaling	High	Yes	Enhances immune responses
**Map2k1**	Kinase (MAPK Pathway)	ERK signaling, immune activation	High	No	Enhances T cell activation
**Fcer1g**	Immune Signaling	Fc receptor signaling, immune activation	High	No	Enhances antigen presentation
**H2-L**	MHC Class I Molecule	Antigen presentation to CD8+ T cells	High	No	Enhances immune response
**Hmgb1**	Nuclear Protein (DAMP)	Chromatin dynamics, inflammation mediator	High	Yes	Strong immune activation[4]
**Lgals3bp**	Immune Modulator	Galectin-related immune signaling	High	Yes	Enhances antigen presentation
**Cdc42**	GTPase Signaling Protein	Actin cytoskeleton regulation, immune modulation	Moderate	No	Modulates immune responses
**Hist2h2aa1**	Histone H2A Variant	DNA damage response, immune modulation	Moderate	Possible	May influence immune recognition
**H2ax**	Histone H2A Variant	DNA damage response, immune recognition	Moderate	Possible	May influence immune response[5]
**H3c1**	Histone H3 Variant	Chromatin regulation, immune modulation	Moderate	No	Possible immune effects
**Tollip**	Immune Regulator	TLR signaling inhibition, immune suppression	Moderate	No	Suppresses immune activation[6]

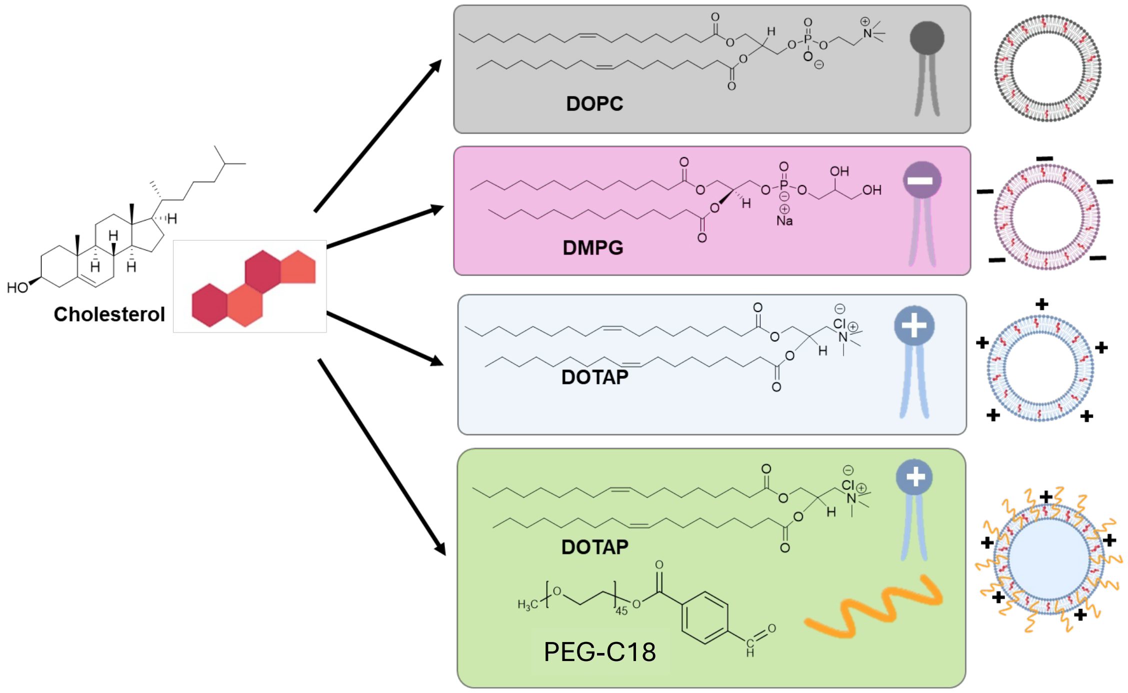

Figure S1. Various formulation of solid lipid nanoparticles.

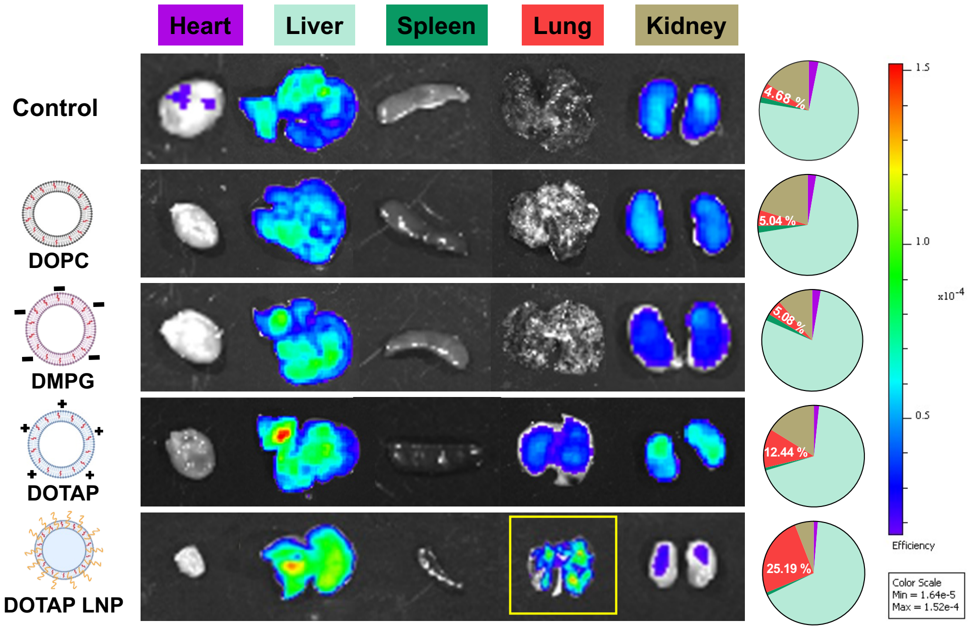

Figure S2. *In vivo* IVIS images of key clearance organs from lung metastasis tumor-bearing mice, captured 24 hours post-treatment with DiI-labeled particles.

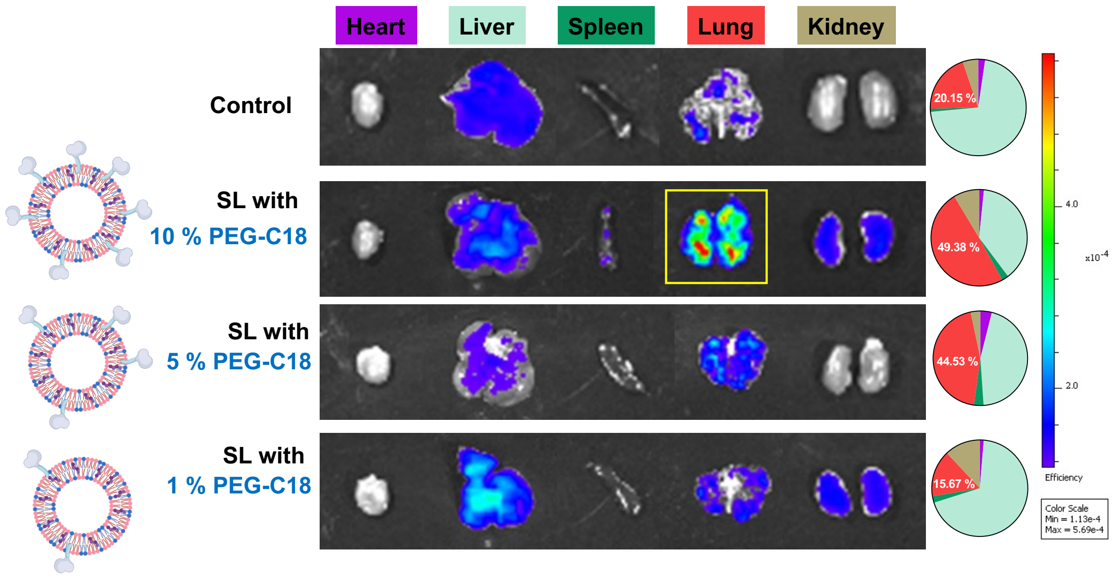

Figure S3. *In vivo* IVIS images of key clearance organs from lung metastasis tumor-bearing mice, captured 24 hours post-treatment with DiI-labeled particles.

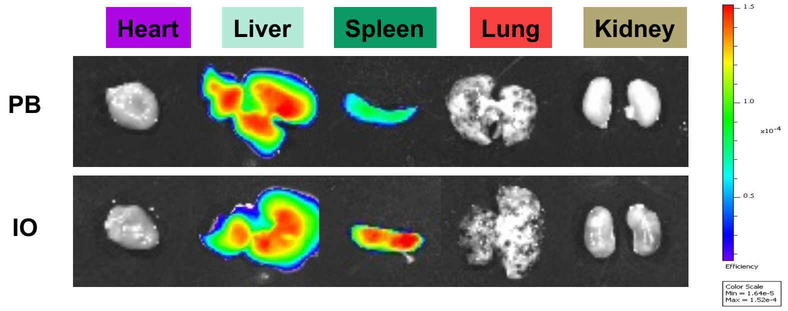

Figure S4. *In vivo* IVIS images of key clearance organs from lung metastasis tumor-bearing mice, captured 24 hours post-treatment with DiI-labeled PB and IO.

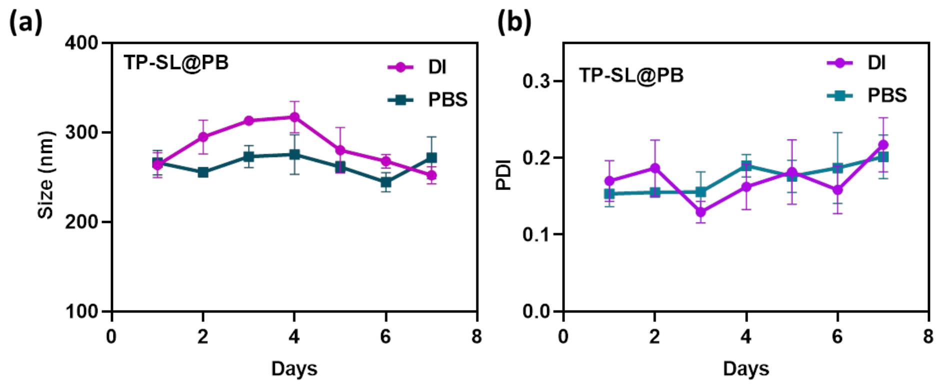

Figure S5. (a) The stability study of hydrated dynamic particle size and (b) polydispersity index (PDI) variation over the time of TP-SL@PB in distilled water or PBS.

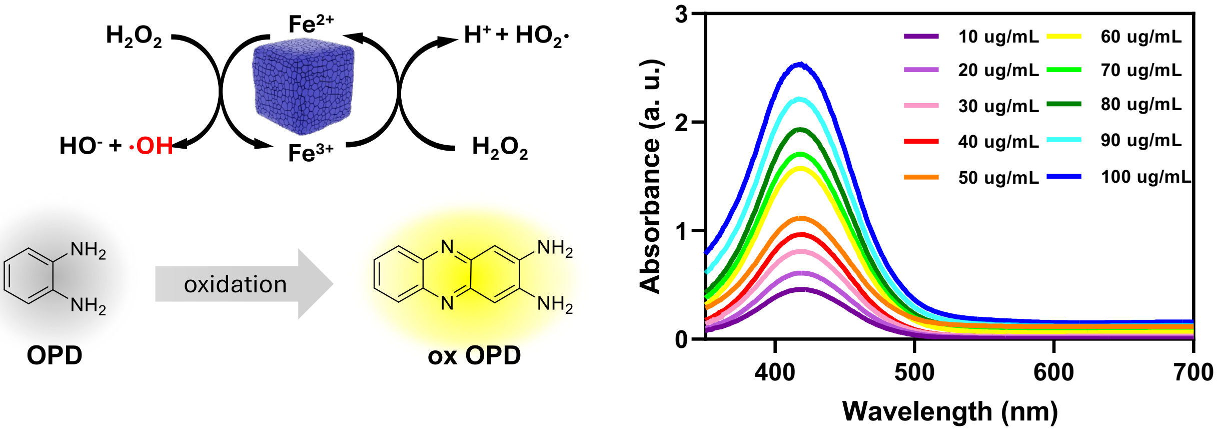

Figure S6. UV-Vis adsorption spectra of the catalyzed oxidation of o-phenylenediamine (OPD) of PB with various concentration of H_2O_2.

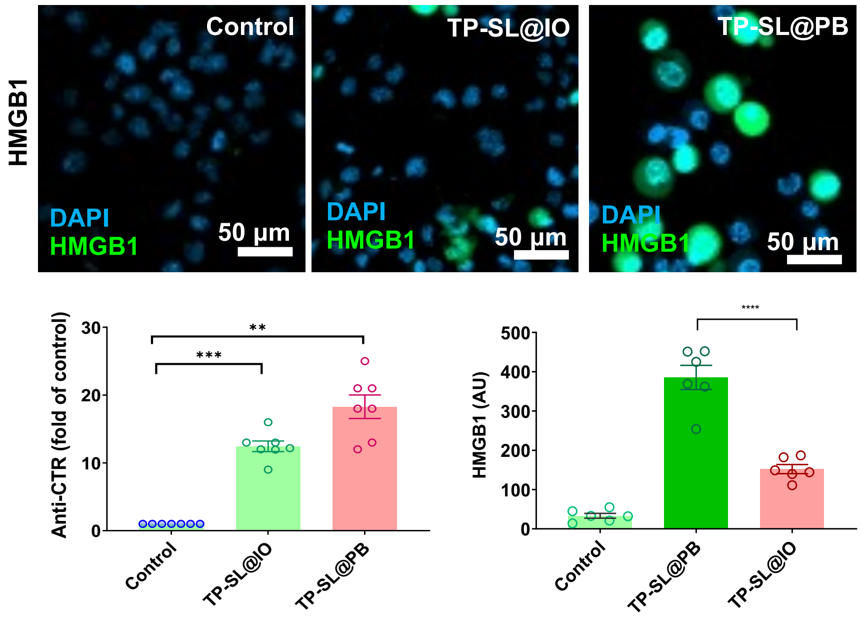

Figure S7. CLSM images of *in vitro* assessments of HMGB1 after treated by TP-SL@PB and TP-SL@IO. Quantitative levels of anti-CRT and HMGB1 are expressed as the percentage of hydroxyl identified in control cells. Statistical significance was assessed using one-way ANOVA. Data represent mean ± SEM, $n = 6$. $^{**}p < 0.01$, $^{***}p < 0.001$.

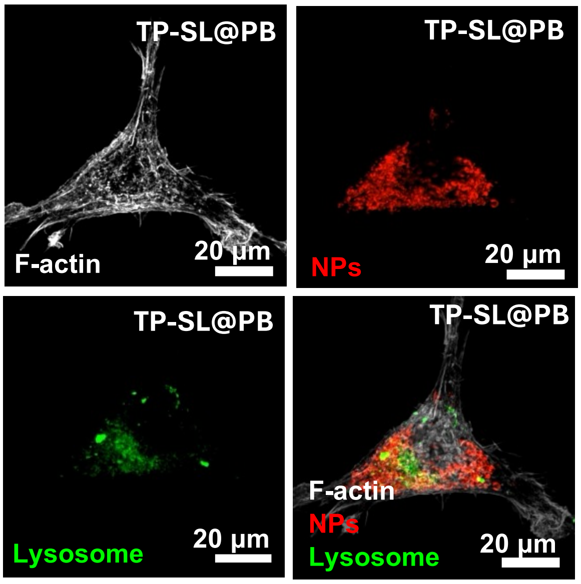

Figure S8. CLSM images of lysosomes of B16F10 cells after treated by TP-SL@PB

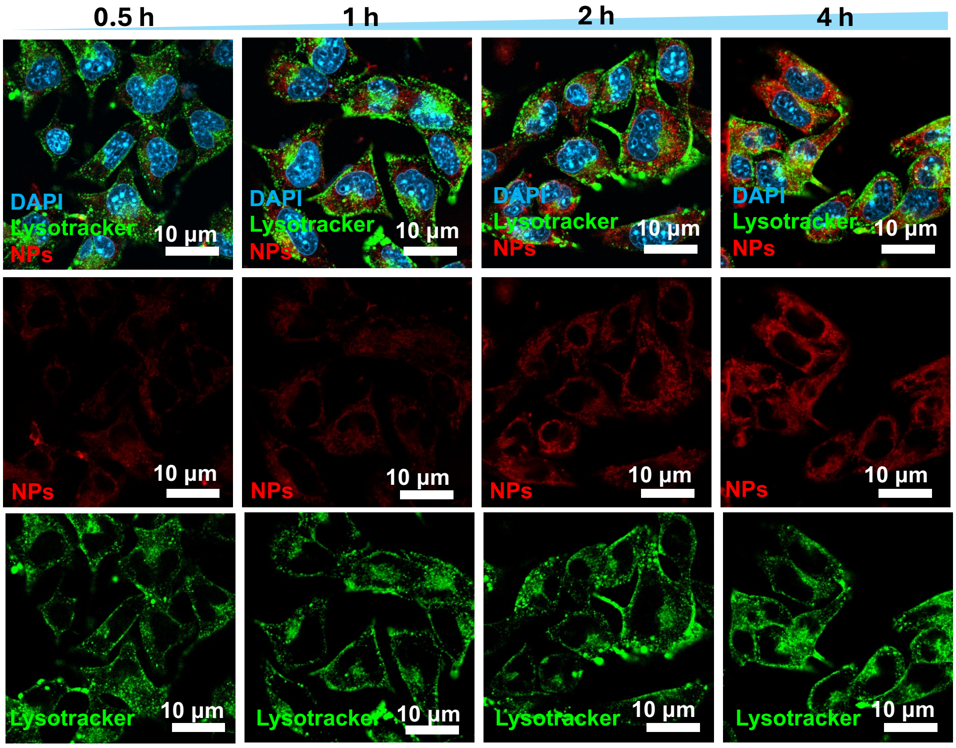

Figure S9. CLSM images of lysosomes in B16F10 cells after incubation with TP-SL@PB for 0.5, 1, 2, and 4 hours.

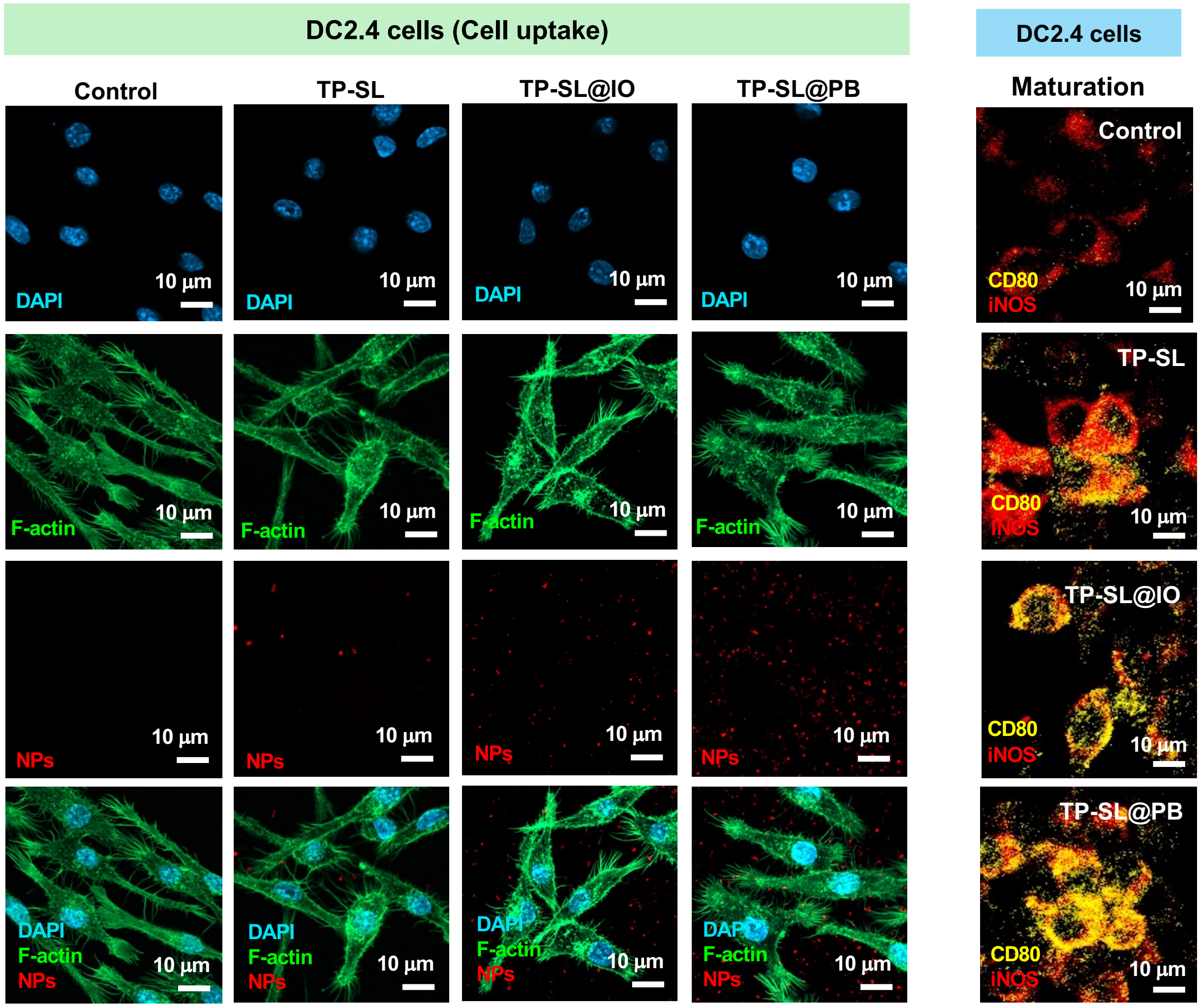

Figure S10. CLSM images of DCs incubated with TP-SL, TP-SL@PB, and TP-SL@IO. Blue, green, and purple represent the nucleus stained with DAPI, the cytoskeleton stained with F-actin, and particles stained with QD, respectively. Additional CLSM images were captured to assess DC maturation following particle incubation.

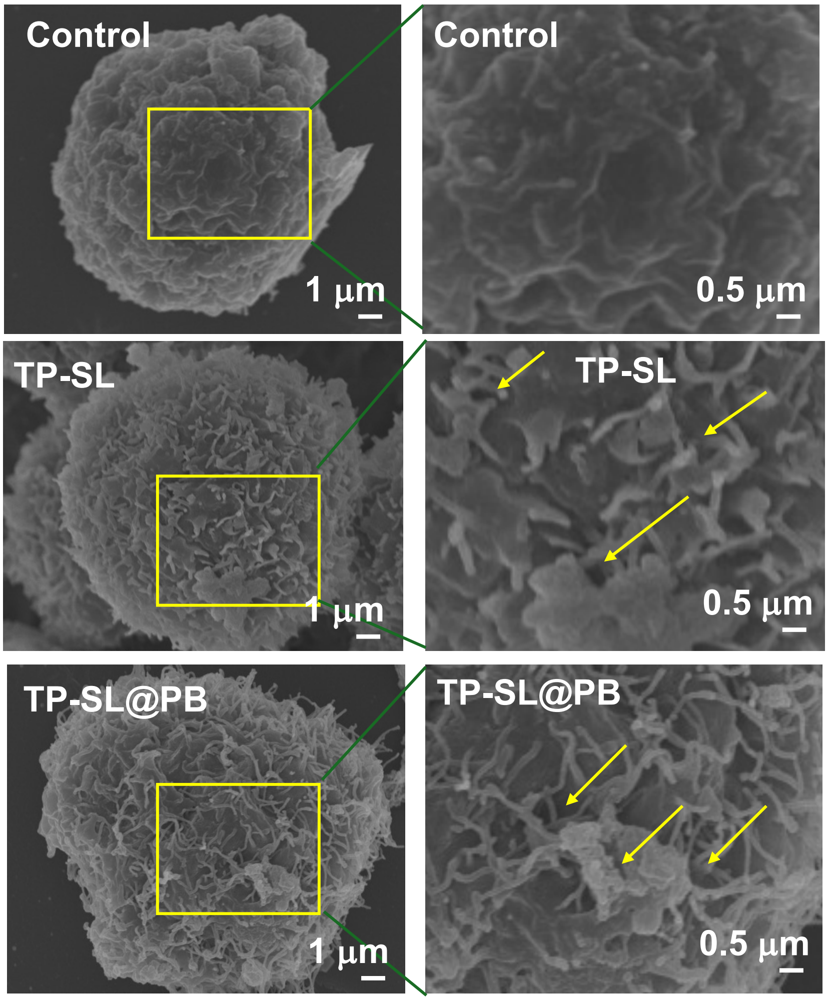

Figure S11. SEM images of B16F10 cells treated by TP-SL and TP-SL@PB.

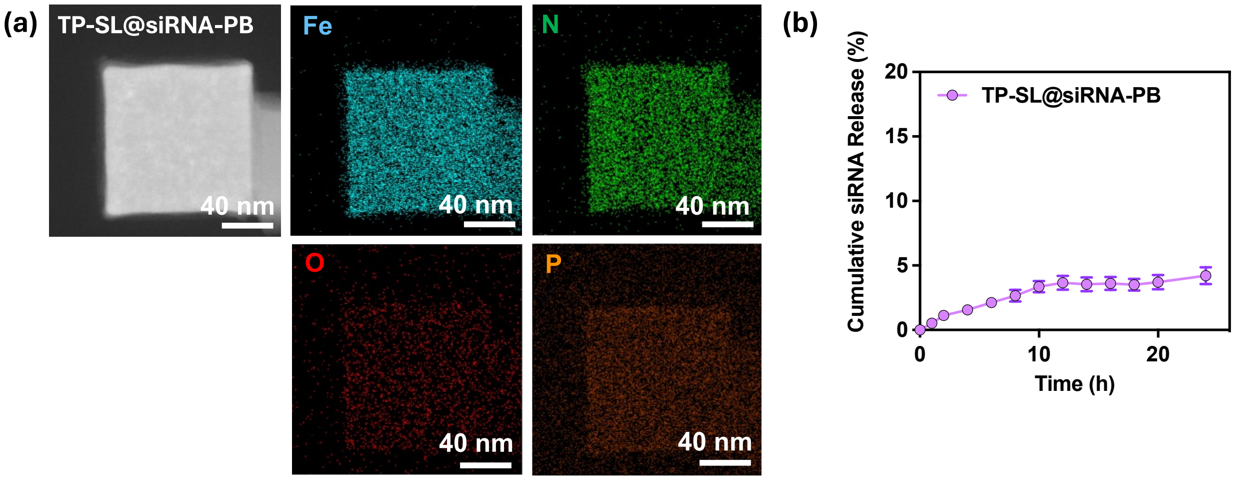

Figure S12. (a) TEM and energy-dispersive X-ray spectroscopy (EDS) mapping images of TP-SL@siRNA-PB. (b) The siRNA release pattern from TP-SL@PB.

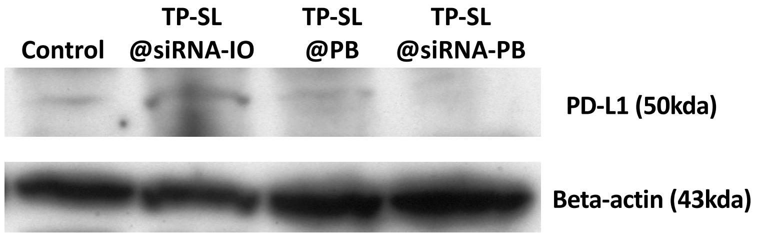

Figure S13. Western blot of PD-L1 expression of B16F10 cells.

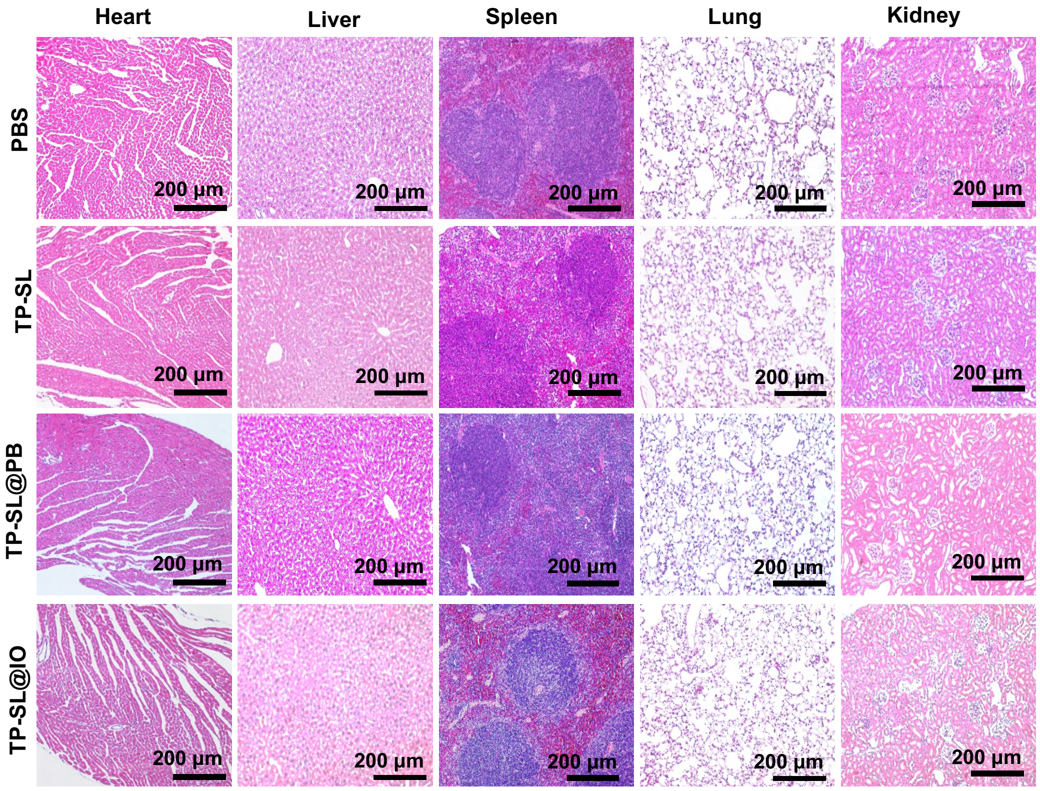

Figure S14. Hematoxylin and eosin (H&E) staining of heart, liver, spleen, lung, and kidney tissues from glioma-bearing mice subjected to various treatment conditions.

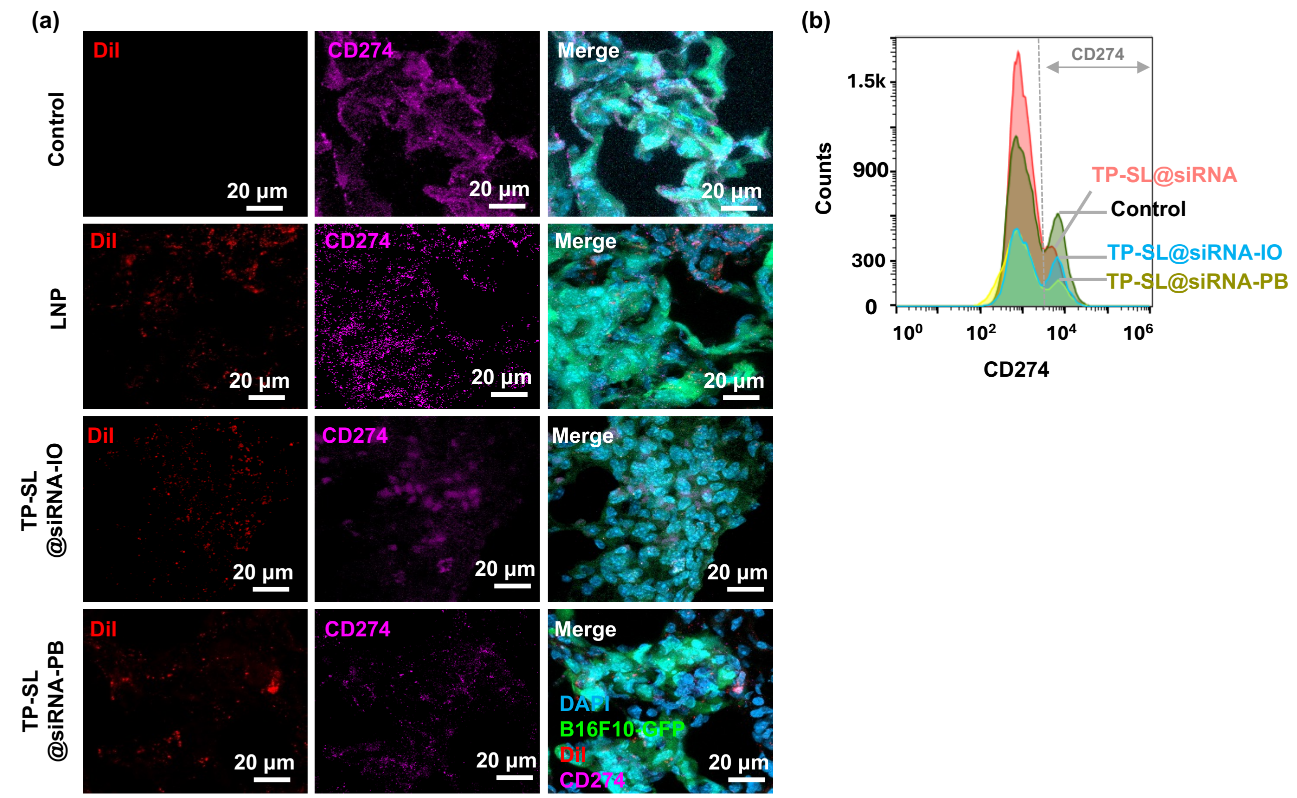

Figure S15. (a) CLSM images and (b) flow cytometry of CD274 expression of lung metastases treated with TP-SL@siRNA-IO and TP-SL@siRNA-PB at 24 h post-injection, respectively.

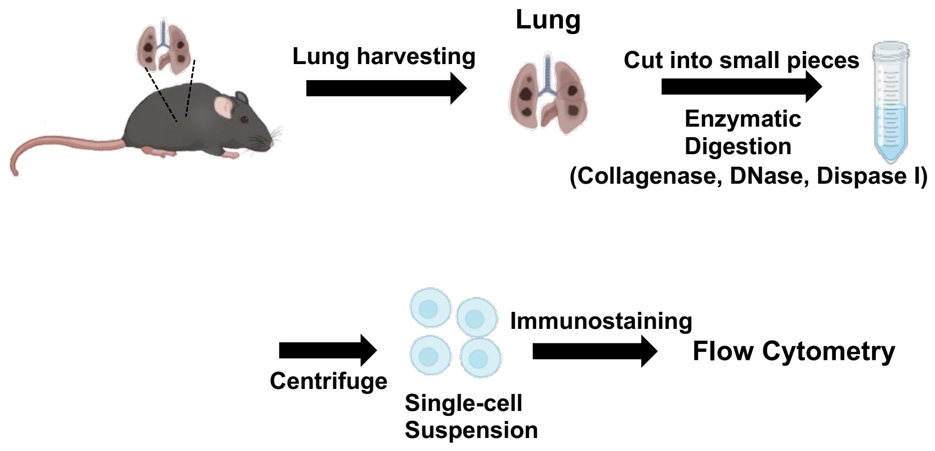

Figure S16. The preparation process for *in vivo* flow cytometry.

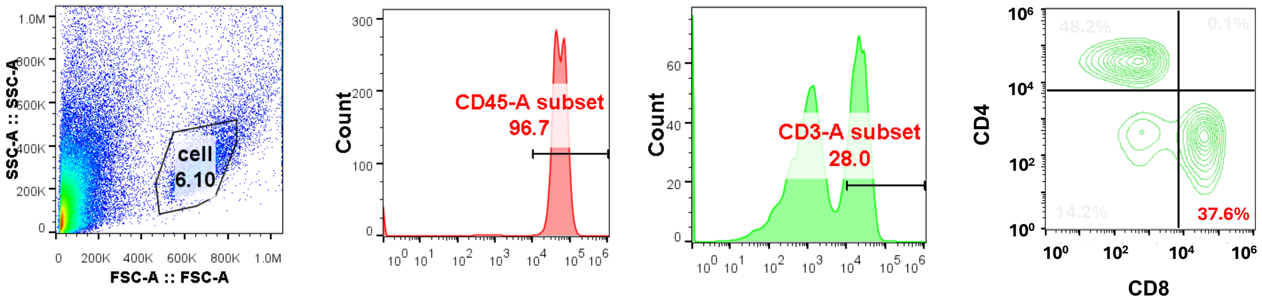

Figure S17. The gating strategy of flow cytometry.

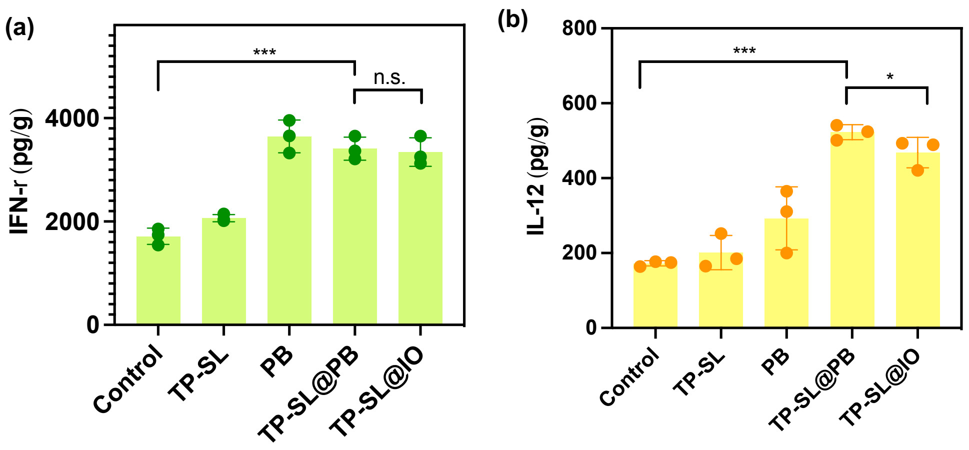

Figure S18. The concentrations of immune factors such as tumor necrosis factor-α (TNF-α), interferon-γ (IFN-γ) and interleukin-12 (IL-12) in brain tissues treated with various samples were quantified using ELISA kits. ($n = 3$; mean ± s.d.; * $p < 0.05$; *** $p < 0.05$; one-way ANOVA with Tukey's multiple comparison test).

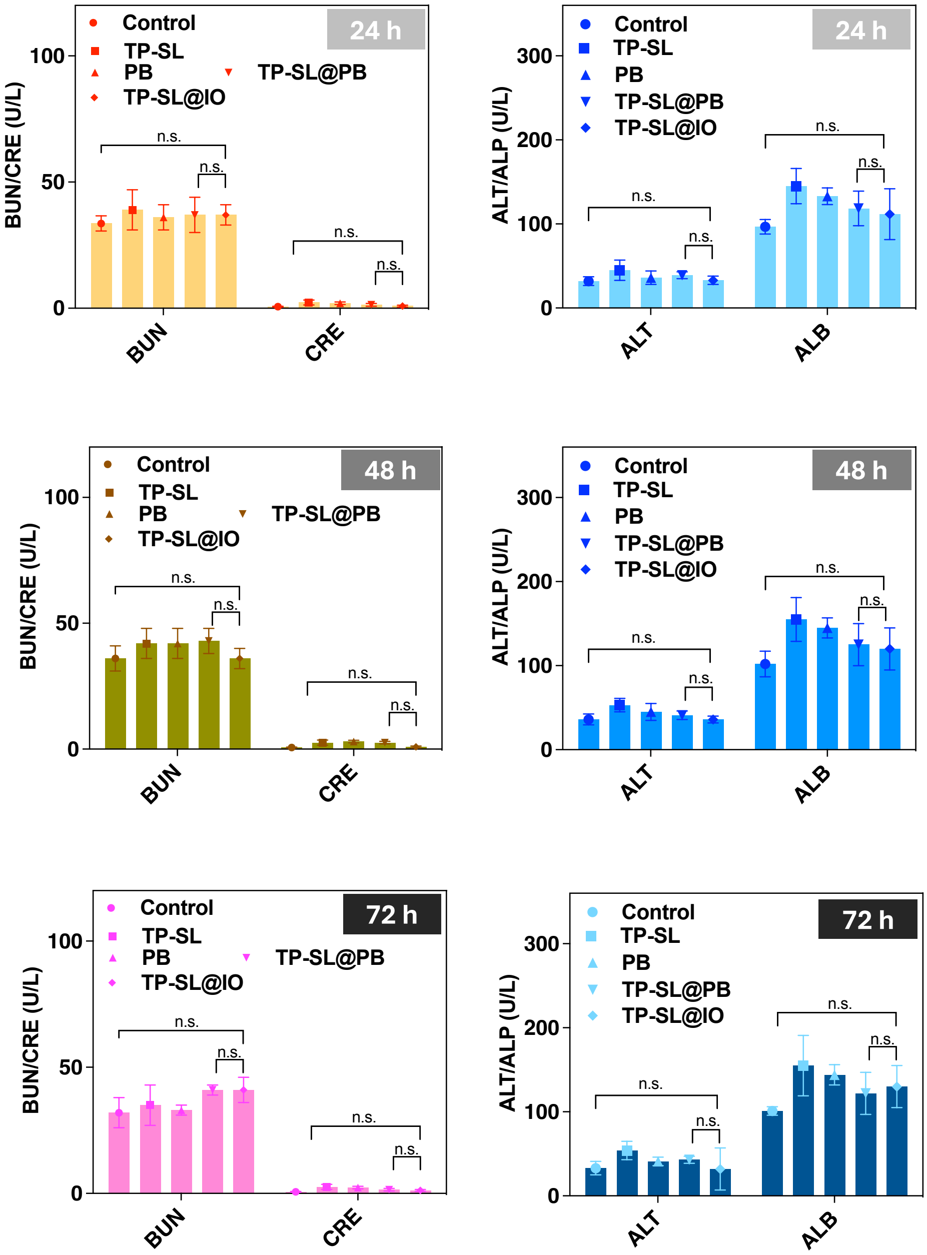

Figure S19. Biochemical indices of liver and kidney after 24 h, 48 h and 72 h of treatment. Statistical significance was assessed using one-way ANOVA. Data represent mean ± SEM, n = 3.

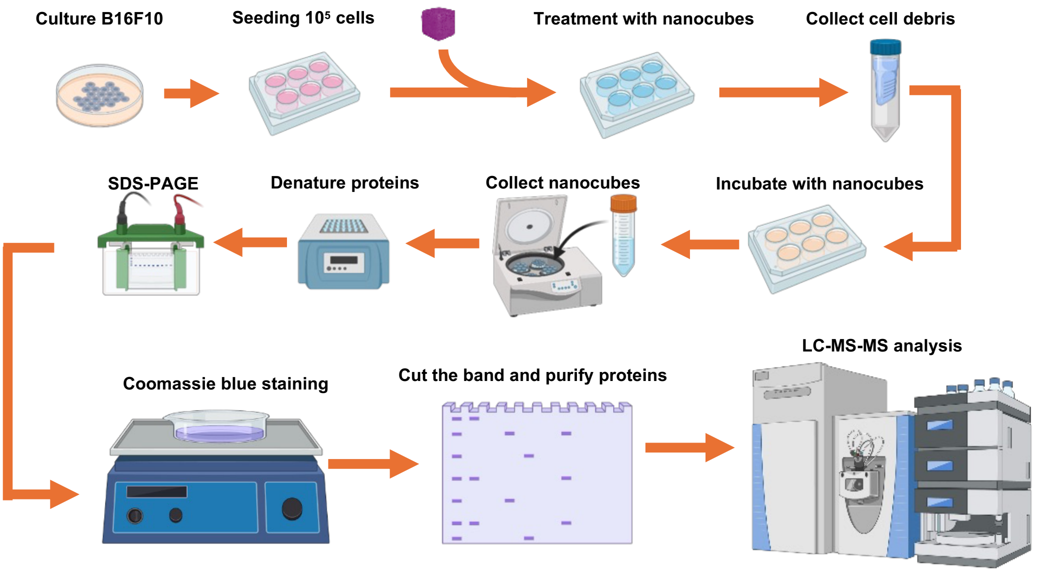

Figure S20. The experimental procedures for analyzing neoantigens and damage-associated molecular patterns (DAMPs) from B16F10 cells following particle treatment. The released antigens were captured using PBA and DON and analyzed by liquid chromatography-mass spectrometry (LC-MS/MS) with an Orbitrap Elite™ hybrid ion trap-Orbitrap mass spectrometer (Thermo Fisher, USA).

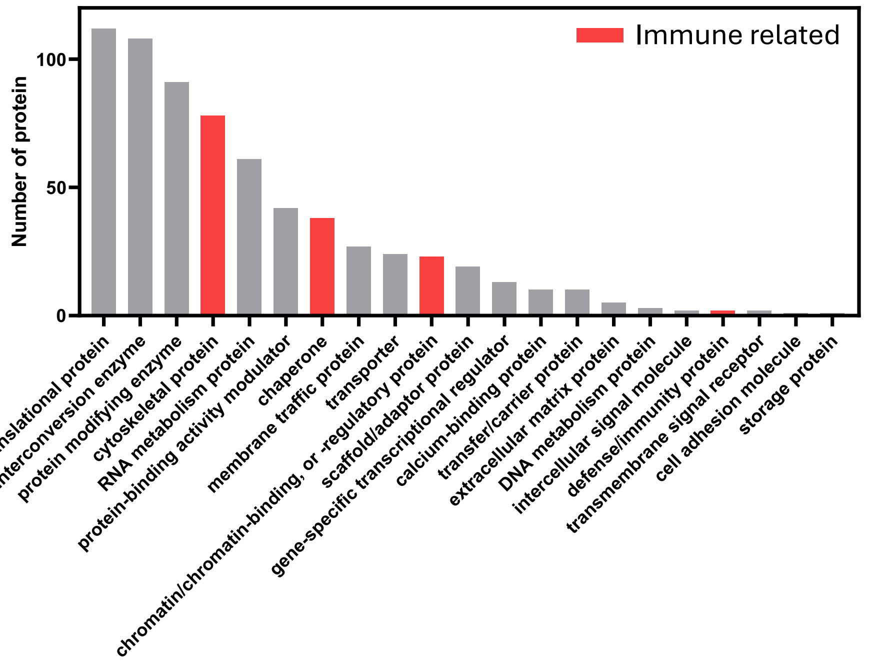

Figure S21. The release of neoantigens and damage-associated molecular patterns (DAMPs) from B16F10 cells following particle treatment was analyzed. A diverse range of related proteins, including 50 highly distinguishable proteins, was identified on TP-SL@PB.

[1] H. Takahashi, K. Yumoto, K. Yasuhara, E. T. Nadres, Y. Kikuchi, L. Buttitta, R. S. Taichman,

K. Kuroda, *Sci. Rep.* **2019**, *9*, 1096.
[2] K. Suzuki, Y. Hiroi, N. Abe-Fukasawa, T. Nishino, T. Shouji, J. Katayama, T. Kageyama, J. Fukuda, *Sci. Rep.* **2022**, *12*, 10815.
[3] K. H. Song, S. J. Oh, S. Kim, H. Cho, H. J. Lee, J. S. Song, J. Y. Chung, E. Cho, J. Lee, S. Jeon, C. Yee, K. M. Lee, S. M. Hewitt, J. H. Kim, S. R. Woo, T. W. Kim, *Nat. Commun.* **2020**, *11*, 562.
[4] M. Park, J. Lim, S. Lee, Y. Nah, Y. Kang, W. J. Kim, *Adv. Mater.* **2025**, *37*, 2417735.
[5] M. Liang, Q. Wang, S. Zhang, Q. Lan, R. Wang, E. Tan, L. Zhou, C. Wang, H. Wang, Y. Cheng, *Adv. Mater.* **2024**, *36*, 2409015.
[6] A. Hu, L. Sun, H. Lin, Y. Liao, H. Yang, Y. Mao, *Signal Transduct. Target .Ther.* **2024**, *9*, 68.
